# An International Continence Society (ICS)/ International Urogynecological Association (IUGA) joint report on the terminology for the assessment and management of obstetric pelvic floor disorders

**DOI:** 10.1007/s00192-022-05397-x

**Published:** 2022-11-28

**Authors:** Stergios K. Doumouchtsis, Renaud de Tayrac, Joseph Lee, Oliver Daly, Joan Melendez-Munoz, Fiona M. Lindo, Angela Cross, Amanda White, Sara Cichowski, Gabriele Falconi, Bernard Haylen

**Affiliations:** 1 Department of Obstetrics and Gynaecology, Epsom and St. Helier University Hospitals NHS Trust, Epsom, UK; 2 St. George’s University of London, London, UK; 3 Laboratory of Experimental Surgery and Surgical Research “N.S. Christeas”, National and Kapodistrian University of Athens, Medical School, Athens, Greece; 4 School of Medicine, American University of the Caribbean, Cupecoy, Sint Maarten; 5 School of Medicine, Ross University, Miramar, FL USA; 6 Nimes University Hospital, Nimes, France; 7 University New South Wales, Sydney, Australia; 8 Western Health, Melbourne, Australia; 9 Hospital Universitari Dr. Josep Trueta, Girona, Spain; 10 Houston Methodist Hospital, Texas A&M University College of Medicine, Houston Methodist Hospital, Houston, TX USA; 11 Middlemore Hospital, Auckland, New Zealand; 12 University of Texas at Austin, Austin, TX USA; 13 Oregon Health & Sciences University, Portland, OR USA; 14 Complex Operative Unit of Gynecology, Fondazione Policlinico Tor Vergata University Hospital, Rome, Italy

**Keywords:** Obstetric pelvic floor disorders, Perineal trauma, Childbirth trauma, Obstetric injuries, Terminology

## Abstract

**Aims:**

The terminology of obstetric pelvic floor disorders should be defined and reported as part of a wider clinically oriented consensus.

**Methods:**

This Report combines the input of members of two International Organizations, the International Continence Society (ICS) and the International Urogynecological Association (IUGA). The process was supported by external referees. Appropriate clinical categories and a sub-classification were developed to give coding to definitions. An extensive process of 12 main rounds of internal and 2 rounds of external review was involved to exhaustively examine each definition, with decision-making by consensus.

**Results:**

A terminology report for obstetric pelvic floor disorders, encompassing 357 separate definitions, has been developed. It is clinically-based with the most common diagnoses defined. Clarity and user-friendliness have been key aims to make it usable by different specialty groups and disciplines involved in the study and management of pregnancy, childbirth and female pelvic floor disorders. Clinical assessment, investigations, diagnosis, conservative and surgical treatments are major components. Illustrations have been included to supplement and clarify the text. Emerging concepts, in use in the literature and offering further research potential but requiring further validation, have been included as an Appendix. As with similar reports, interval (5–10 year) review is anticipated to maintain relevance of the document and ensure it remains as widely applicable as possible.

**Conclusion:**

A consensus-based Terminology Report for obstetric pelvic floor disorders has been produced to support clinical practice and research.

## Introduction

Obstetric pelvic floor disorders encompass a range of anatomical and functional changes associated with pregnancy and childbirth. Pelvic floor trauma refers to injuries of the different anatomical structures of the pelvic floor and commonly occurs at the time of the first vaginal childbirth. Perineal and vaginal as well as anal sphincter trauma following delivery are the most commonly described types of obstetric trauma. However, different aspects of the pelvic floor may become affected through tissue rupture, compression, stretching, with associated nerve, muscle and connective tissue damage.

Perineal trauma affects millions of women with an incidence of over 91% in nulliparous women and over 70% in multiparous women [1] and has a potentially significant impact on daily activities, psychological wellbeing, sexual function and overall quality of life [2]. Anal sphincter injury is clinically diagnosed in 1%–11% of women following vaginal delivery [3]. Recently documented increases in the reported incidence have been attributed to improved training and awareness around anal sphincter injury [3, 4].

No single document encompasses all elements required for classification, diagnosis and treatment of obstetric pelvic floor disorders. Such a report requires a full outline of the terminology for the different types of obstetric trauma, symptoms, signs, clinical assessments, imaging and functional investigations, the most common diagnoses, prediction, prevention, and management.

The aim of this Working Group was to develop a Terminology Report as a definitional document, collating the definitions of those terms, related to obstetric pelvic floor disorders. This document will include definitions of perineal and pelvic floor trauma and associated disorders. Definitions of pelvic floor disorders that occur during pregnancy and up to 12 months postpartum will be considered for inclusion, for the purpose of this document. The rationale for this choice was based on evidence around the effect of pregnancy, childbirth and postnatal factors on the pelvic floor and the natural history of pelvic floor function recovery postnatally as well as possible influences of breastfeeding.

As obstetric pelvic floor disorders encompass a wide range of anatomical and functional changes, it could potentially give rise to all definitional terms involving the entire pelvic floor. Important definitions already defined in existing terminology documents are acknowledged and presented where relevant. Explanatory notes on definitions have been expanded, where applicable as footnotes. Emphasis has been on the inclusion of terms in the relevant peer-reviewed literature. The aim is to assist clinical practice as well as research by developing a repository of appropriately selected and defined terms that will advise communication among professionals at different levels in the area of pelvic floor clinical practice as well as obstetric practice and research.

**Disorder** is a disruption of normal physical function, a disease or abnormal condition. (NEW).

**Trauma** is defined as physical injury or a deeply distressing or disturbing experience. (NEW).

**Injury** is a form of physical trauma that refers to impact on the relevant anatomical tissues and structures. (NEW).

**Obstetric pelvic floor disorders** refer to effects of pregnancy and childbirth on anatomy and function of the pelvic floor appearing up to 12 months postpartum. (NEW).

**Postpartum period** refers to the period that begins upon the delivery of the infant to 12 months after the delivery. (NEW).

The methodology followed for the selection of terms and the process of consensus on definitions is presented in Appendix 1.

This Terminology Report is inherently and appropriately a definitional document, collating the definitions of those terms, i.e. “words used to express a defined concept in a particular branch of study” [5] concerning obstetric pelvic floor disorders. Existing, new and revised terms have been included.

In line with all the other joint ICS-IUGA female-specific terminology reports, every effort has been made to ensure this Report is:

1. **User-friendly:** It should be able to be understood by all clinical and research users.
2. **Clinically-based:** Symptoms, signs and validated assessments/investigations should be presented for use in forming workable diagnoses for obstetric pelvic floor disorders and associated dysfunctions. Sections 1–4 will address symptoms, signs, investigations, diagnoses for associated dysfunctions. Sections 5 and 6 will list the terminology for prediction, prevention and evidence-based management for obstetric pelvic floor disorders.
3. **Origin:** Where a term’s existing definition (from one of multiple sources used) is deemed appropriate, that definition will be included and duly referenced. A large number of terms in obstetric pelvic floor disorders, have now become generic because of their long-term use, as apparent by their listing in medical dictionaries.
4. **Able to provide explanations:** Where a specific explanation is deemed appropriate to describe a change from earlier definitions or to qualify the current definition, this will be included as an addendum to this paper. Wherever possible, evidence-based medical principles will be followed (see Table 1).

**Table 1:** Total, new and changed definitions (compared with previous ICS Reports)

Section	New definitions / descriptors	Changed definitions / descriptors*	Unchanged	Total
Introduction & symptoms	50	1	30	81
Signs	24	3	48	75
Investigations	14	n/a	33	47
Diagnoses	48	1	29	78
Prediction and prevention	36	n/a	11	47
Management	11	n/a	18	29
Total	181(51%)	5 (1%)	169 (48%)	357

Previous ICS and ICS/IUGA Terminology Reports that will be referenced are the 2010 IUGA-ICS Joint Terminology Report on Female Pelvic Floor Dysfunction [6], 2019 the International Continence Society (ICS) report on the terminology for adult male lower urinary tract and pelvic floor symptoms and dysfunction [7], 2016 ICS-IUGA Joint Terminology Report on Female Pelvic Organ Prolapse [8], 2017 ICS-IUGA Joint Terminology Report on Female Anorectal Dysfunction [9] and 2020 ICS Report on the Terminology for Female Pelvic Floor Fistulas [10].

It is suggested that acknowledgment of these standards in written publications related to obstetric pelvic floor disorders, be indicated by a footnote to read as follows: “Methods, definitions and units conform to the standards jointly recommended by the International Continence Society and International Urogynecological Association except where specifically noted”.

## Symptoms

### Symptom

Any morbid phenomenon or departure from the normal in structure, function or sensation, experienced by the woman and indicative of disease or a health problem [11]. _FN1.1_

### Lower urinary tract symptoms

#### Storage symptoms

Lower urinary tract symptoms occurring during the bladder storage phase [7].

##### Bladder filling (sensory) symptoms:

Abnormal sensations experienced during bladder filling [7].

##### Urinary incontinence (UI):

Complaint of involuntary loss of urine experienced during the bladder storage phase [7].

##### Stress urinary incontinence (SUI):

Complaint of involuntary loss of urine on effort or physical exertion including sporting activities, or on sneezing or coughing [7].

##### Urgency urinary incontinence (UUI):

Complaint of involuntary loss of urine associated with urgency [7].

##### Mixed urinary incontinence (MUI):

Complaints of both stress and urgency urinary incontinence, i.e. involuntary loss of urine associated with urgency and also with effort or physical exertion including sporting activities or on sneezing or coughing [7].

##### Coital urinary incontinence:

Complaint of involuntary urine loss during or after coitus. This symptom might be further divided into that occurring with penetration and that occurring at orgasm [12].

##### Nocturnal enuresis:

Complaint of involuntary voiding that occurs at night during the main sleep period (i.e. bedwetting) [13].

##### Urgency:

Complaint of a sudden, compelling desire to pass urine which is difficult to defer [6, 7, 14, 15].

##### Coital urinary urgency:

Feeling of urgency to void during vaginal intercourse [12].

##### Nocturia:

Complaint that urine is passed during the main sleep period. Having woken to pass urine for the first time, each urination must be followed by sleep or the intention to sleep. This should be quantified using a bladder diary [7, 13].

##### Voiding symptoms:

Lower urinary tract symptoms experienced during the voiding phase (experienced during micturition) [7].

##### Hesitancy:

Complaint of a delay in initiating voiding (when the individual is ready to pass urine) [7].

##### Slow (urinary) stream:

Complaint of a urinary stream perceived as overall slower than previous performance or in comparison with others [7].

##### Intermittency (intermittent stream):

Complaint of urine flow that stops and starts on one or more occasions during one voiding episode [7].

##### Straining to void:

Complaint of the need to make an intensive effort to either initiate, maintain or improve voiding or the urinary stream [7].

##### Spraying (splitting) of urinary stream:

Complaint that the urine passage is a spray or split rather than a single directional stream [7].

##### Position-dependent voiding:

Complaint of having to take specific positions to be able to void spontaneously or improve bladder emptying, e.g. leaning forwards or backwards on the toilet seat or voiding in a semi-standing position [6].

##### Dysuria:

Complaint of pain, burning or other discomfort during voiding. Discomfort may be intrinsic to the lower urinary tract (e.g. bladder or urethra), external or referred from other adjacent similarly innervated structures e.g. lower ureter [7].

#### Post micturition symptoms

##### Need to immediately re-void (“encore” or “double” voiding):

Complaint that further micturition is necessary soon after passing urine (cessation of flow) [7].

##### Post-micturition leakage (incontinence):

Complaint of a further involuntary passage of urine following the completion of voiding [6].

### Anorectal and defecatory symptoms

#### Feeling of incomplete bowel evacuation

Complaint that the rectum does not feel empty after defecation. May be accompanied by the desire to defecate again [7].

#### Straining to defecate

Complaint of the need to make an intensive effort, by abdominal straining, or to use abdominal massage to either initiate, maintain or improve defecation [7, 16, 17].

#### Manual defecatory assistance

##### Splinting:

Support perineum or buttocks manually (usually with thumb or fingers) to assist in evacuation of stool content [9, 18].

##### Splinting/digitation:

Complaint of the need to digitally manipulate the vaginal wall or to otherwise apply manual pressure e.g. to the vagina or perineum (splinting), or to the vagina or rectum (digitation) to assist defecation. (NEW). [8, 14] _FN1.2_.

#### Fecal (rectal) urgency

Complaint of a sudden compelling desire to defecate that is difficult to defer [9, 18].

#### Post-defecatory soiling

Complaint of soiling (passing of stool into clothing) occurring after defecation [7].

### Pelvic organ prolapse symptoms

#### Vaginal bulging

Complaint of a “bulge”, lump or “something coming down” or “falling out” through the vaginal introitus. The woman may state, or not, she can either feel the bulge by direct palpation or see it, perhaps aided with a mirror [8].

#### Pelvic pressure

Complaint of increased heaviness or dragging (pain or discomfort) in the suprapubic area and/or pelvis [8] (CHANGED).

#### Splinting/Digitation due to POP

Complaint of the need to digitally replace the prolapse or to otherwise apply manual pressure, e.g. to the vagina or perineum (splinting), or to the vagina or rectum (digitation) to assist voiding or defecation [8].

#### Urethral prolapse

Complaint of “lump” at the external urethral meatus [8].

#### Rectal prolapse

Complaint of a “bulge” or “something coming down” towards or through the anus/rectum. The woman may state she can either feel the bulge by direct palpation or see it perhaps aided with a mirror [9, 18]. _FN1.3_

### Obstetric pelvic floor trauma related pelvic symptoms

Obstetric pelvic floor trauma may cause anatomical changes to pelvic floor musculature, connective tissue, nerves and vulvo-vaginal surrounding organs, bladder and rectum most commonly. This can lead to abnormal function, most commonly storage and voiding symptoms affecting the bladder and bowel (NEW).

### Obstetric pelvic floor trauma related urinary tract symptoms _FN1.4_

#### Pregnancy and postpartum storage symptoms

##### Pregnancy associated urinary incontinence:

Complaint of involuntary loss of urine during pregnancy (NEW). _FN1.5_

##### Postpartum urinary incontinence (PPUI):

Complaint of involuntary loss of urine experienced during the postpartum period and up to 12 months after delivery (NEW). [7]. _FN1.6_

##### Postpartum stress urinary incontinence (PPSUI) (symptom):

Complaint of involuntary loss of urine on effort or physical exertion including sporting activities, or on sneezing or coughing experienced for the first time during the postpartum period and up to 12 months after delivery (NEW) [7].

##### Postpartum urgency urinary incontinence (PPUUI):

Complaint of involuntary loss of urine associated with the sensation of a sudden, compelling desire to pass urine which is difficult to defer experienced for the first time during the postpartum period and up to 12 months after delivery (NEW).

##### Postpartum mixed urinary incontinence (PPMUI):

Complaints of both stress and urgency urinary incontinence, i.e. involuntary loss of urine associated with urgency and also with effort or physical exertion including sporting activities or on sneezing or coughing experienced for the first time during the postpartum period and up to 12 months after delivery (NEW) [7].

##### **Postpartum coital** urinary incontinence:

Complaint of involuntary urine loss during or after coitus experienced for the first time during the postpartum period and up to 12 months after delivery. This symptom might be further divided into that occurring with penetration and that occurring at orgasm (NEW) [12].

##### Postpartum daytime urinary frequency:

Complaint that voiding occurs more frequently during waking hours than previously deemed normal by the woman during the postpartum period and up to 12 months after delivery (NEW) [7].

#### Postpartum voiding symptoms

Lower urinary tract symptoms related to the voiding phase that appeared during the postpartum period and up to 12 months after delivery [7] (NEW).

##### Postpartum de novo urinary retention:

Complaint of inability to empty the bladder as before (to distinguish from predelivery/pre-pregnancy difficulties), despite the ability to pass some urine during the postpartum period and up to 12 months after delivery. (NEW). _FN1.7_

##### Postpartum hesitancy:

Complaint of inability to initiate micturition during the postpartum period and up to 12 months after delivery. (NEW).

##### Postpartum voiding difficulty:

Complaint of inability to empty the bladder (regardless of whether emptying is complete or not) _FN1.8_ in relation to:

- characteristics of urination flow (spraying, slow stream etc.…)
- how urination takes place (for example regarding the position to be maintained during micturition, whether or not external pressure on the abdominal or vaginal walls is applied, use of a catheter, etc.) and
- increased or reduced voiding interval (NEW). _FN1.9_

##### Postpartum urinary retention (PPUR):

C [7] (NEW). _FN1.9_

##### Postpartum incomplete (bladder) emptying:

Complaint that the bladder does not feel empty, after voiding has ceased, during the postpartum period and up to 12 months after delivery (NEW) [7].

##### Postpartum splinting to micturate:

Complaint of the need to digitally support perineum or buttocks manually (usually with thumb or fingers) to assist voiding (micturition), eventually manually reducing the prolapse, first experienced during postpartum period and up to 12 months after delivery (NEW).

### Pregnancy and postpartum associated defecatory or post-defecatory symptoms

Symptoms experienced during or following the act of defecation during pregnancy, the postpartum period and up to 12 months after delivery [7] (NEW).

#### Postpartum constipation

Complaint that bowel movements are infrequent and/or incomplete and/or there is a need for frequent straining or manual assistance to defecate (Rome IV criteria [19, 20]) during the postpartum period and up to 12 months after delivery (NEW). _FN1.10,FN1.11_

#### Postpartum post-defecatory pain

Complaint of pain occurring during or after defecation during the postpartum period and up to 12 months after delivery (NEW).

#### Postpartum post-defecatory rectal bleeding

Complaint of rectal bleeding occurring after defecation during the postpartum period and up to 12 months after delivery (NEW).

#### Postpartum anal incontinence (symptom)

Complaint of involuntary loss of flatus or feces during the postpartum period and up to 12 months after delivery (NEW).

##### Postpartum fecal incontinence:

Complaint of involuntary loss of feces (solid and/or liquid) during the postpartum period and up to 12 months after delivery [7] (NEW).

##### Postpartum flatal incontinence:

Complaint of involuntary loss of flatus (gas) during the postpartum period and up to 12 months after delivery [9, 18] (NEW).

##### Postpartum coital anal incontinence:

Fecal or flatal incontinence occurring with vaginal intercourse during the postpartum period and up to 12 months after delivery [12] (NEW).

##### Postpartum passive fecal (insensible) incontinence:

Fecal soiling without sensation or warning or difficulty wiping clean during the postpartum period and up to 12 months after delivery [9] (NEW).

##### Postpartum overflow fecal incontinence:

Complaint of involuntary loss of stool due to an overfull rectum or fecal impaction during the postpartum period and up to 12 months after delivery [9, 18] (NEW).

##### Postpartum fecal urgency incontinence:

Complaint of involuntary loss of feces associated with the sensation of a sudden, compelling desire to pass feces which is difficult to defer experienced for the first time during the postpartum period and up to 12 months after delivery (NEW).

##### Postpartum anal mucus incontinence:

Complaint of the loss of mucus per rectum during the postpartum period and up to 12 months after delivery (NEW).

##### Postpartum double incontinence:

Complaint of both anal incontinence and urinary incontinence during the postpartum period and up to 12 months after delivery (NEW).

### Ante/Postpartum prolapse symptoms

A departure from normal sensation, structure or function, experienced by the woman about the position of her pelvic organs during pregnancy or in the postpartum period and up to 12 months after delivery (NEW). _FN1.1_

### Postpartum bleeding, discharge, infection

Complaint of abnormal vaginal and/or vulvo-perineal bleeding, mucus/pus discharge during the postpartum period and up to 12 months after delivery (NEW).

### Obstetric pelvic floor trauma related sexual dysfunction symptoms [6, 12, 21]

#### Postpartum dyspareunia

Complaint of persistent or recurrent pain or discomfort associated with attempted or complete vaginal penetration during the postpartum period and up to 12 months after delivery (NEW).

#### Postpartum vaginal laxity

Feeling of vaginal looseness during the postpartum period and up to 12 months after delivery [12] (NEW).

#### Postpartum obstructed intercourse

New onset difficulty or obstruction of vaginal intercourse that occurs during the postpartum period and within 12 months after delivery (NEW).

#### Postpartum anorgasmia or difficulty in achieving orgasm

Complaint of lack of orgasm during the postpartum period and within 12 months after delivery; the persistent or recurrent difficulty, delay in or absence of attaining orgasm following sufficient sexual stimulation and arousal, which causes personal distress (NEW).

#### Postpartum decreased arousal

Persistent or recurrent inability to achieve or maintain sexual excitement during the postpartum period and within 12 months after delivery. This may be expressed as lack of excitement, lack of lubrication, lack of vaginal and clitoral engorgement, or lack of expression of other somatic responses (NEW).

#### Postpartum decreased libido or sexual desire

Absent or diminished feelings of sexual interest or desire, absent sexual thoughts or fantasies, and a lack of responsive desire during the postpartum period and up to 12 months after delivery. Motivations (here defined as reasons/incentives) for attempting to become sexually aroused are scarce or absent. The lack of interest is considered to be beyond the normative lessening with lifecycle and relationship duration (NEW).

#### Postpartum reduced vulvo-vaginal sensation (symptom)

Reduced vulvo-vaginal sensation to touch, pressure, vibration or temperature during the postpartum period and up to 12 months after delivery (NEW).

### Obstetric pelvic floor trauma related pain symptoms

#### Postpartum perineal pain

Complaint of pain felt between the posterior fourchette (posterior lip of the vaginal introitus) and the anus during the postpartum period and up to 12 months after delivery (NEW).

#### Postpartum vulval pain

Complaint of pain felt between the posterior fourchette (posterior lip of the vaginal introitus) and the mons pubis limited by the inner thigh fold and including labial, clitoral and periurethral pain during the postpartum period and up to 12 months after delivery (NEW).

#### Postpartum pudendal pain

Complaint of pain, pressure or discomfort referred to pubic symphysis, labia majora and minora, inferior third of the vagina, urethral meatus, anus, perianal area, lower third of the rectum and buttock (possible inflammation or entrapment of the pudendal nerve and involving its dermatome) during the postpartum period and up to 12 months after delivery [7] (NEW).

#### Postpartum pubic pain

Complaint of pain in the symphysis pubic area during the postpartum period and up to 12 months after delivery (NEW).

#### Postpartum coccygeal pain

Complaint of pain, pressure or discomfort felt in the coccygeal region during the postpartum period and up to 12 months after delivery [7] (NEW).

#### Postpartum pelvic joint, ligament or bone pain

Complaint of joint and/or bony pain described at level of pelvic and perineal area during the postpartum period and up to 12 months after delivery (NEW).

#### Obstetric pelvic girdle syndrome

A symptom syndrome which involves pain in all three pelvic joints or unilateral/bilateral sacroiliac joint pain, which may also occur before or after childbirth [22] (NEW).

**Footnotes for Section** **Symptoms**

**1.1:** Symptoms are generally worse in situations when gravity might make the prolapse worse (e.g. after long periods of standing or exercise) and better when gravity is not a factor e.g. lying supine. Symptoms may also be more noticeable at times of abdominal straining e.g. defecation or bearing down [6]. Straining is a different manoeuvre to Valsalva and results in different effects on pelvic floor activity and pelvic organ descent [16]. It has been proposed that Valsalva and straining have different PFM activation patterns. The pelvic floor is stiffer with Valsalva resulting in better bladder neck (BN) support whereas straining leads to more puborectalis and bladder neck descent [17].

Postpartum prolapse, urinary and in general pelvic floor symptoms are often self-limiting and may resolve after the postpartum period, however in some cases they may persist [23–25].

We acknowledge that definitions of pelvic floor symptoms are presented extensively in other relevant terminology reports. In order to provide a comprehensive inventory of commonly encountered pelvic floor symptoms in pregnancy and postpartum period, we provide a selected list of generic pelvic floor definitions in addition to symptoms and their definitions that have specific characteristics in pregnancy and postpartum period for comprehensiveness and ease of reference. As health care providers outside the area of pelvic floor (urogynecology, female urology) such as obstetricians, midwives etc. may find this report useful without necessarily having access to other terminology reports, we decided to allow such overlaps.

**1.2:** Manipulation of the vaginal wall also includes manual repositioning, if prolapsed.

**1.3:** Anus does not prolapse in these patients, only rectum. Rectal prolapse based is differentiated based on the degree of rectal intussusception in relation to the fixed landmark of anus as intrarectal, intraanal and external. These may not be seen or felt by direct palpation till they are external. The others present with symptoms. Intrarectal prolapse presents with symptoms of obstructed defecation and intraanal prolapse presents with both obstructed defecation and fecal incontinence.

**1.4:** In the postpartum period, voiding symptoms or other LUTS can be secondary to regional anesthesia, indwelling transurethral catheterization or overdistention bladder injury.

**1.5:** It was decided not to change the definition of the urinary symptoms experienced by women during pregnancy, considering their incidence, duration and, last but not least, the possibility of hindering documentation and reporting in clinical practice.

**1.6:** Pregnancy and childbirth cause temporary and sometimes permanent changes to the anatomical structures of the pelvic floor. Sequelae on pelvic organ function can be unpredictable both in the short and long term. Epidemiological and comparative anatomy studies suggest that postpartum pelvic floor dysfunction (for example urinary incontinence) could have a different etiopathogenesis than those experienced in a more advanced age [26].

**1.7:** If the woman experiences voiding difficulties and/or small frequent voids during the first hours post childbirth, the post-void residual volume should always be investigated. This should be performed even in the absence of specific symptoms if voiding interval is longer than 3–4 h, especially after epidural/regional anesthesia.

**1.8:** Voiding difficulty has been defined as an abnormally slow or incomplete voiding [27].

Voiding dysfunction is defined by symptoms and urodynamic investigations as abnormally slow and/or incomplete micturition, based on abnormally slow urine flow rates and or abnormally high post-void residuals, ideally on repeated measurement to confirm abnormality. Pressure-flow studies can be required to determine the cause of voiding dysfunction [8].

**1.9:** Postpartum urinary retention (PPUR) is usually:

- an acute condition due to trauma or inflammatory/infection changes to the lower urogenital tract associated with spontaneous or operative (vaginal or abdominal) delivery;
- characterized by absent or reduced woman’ perceived bladder sensation;
- may be associated with acute pain in the initial phase of retention;
- may also be associated with small frequent voids that do not sufficiently empty the bladder;
- resolves, if properly recognized and treated, in a few hours or days.

For the management of PPUR, optimal management of bladder emptying is essential both during labor and in the postpartum period. Timed voiding and, possibly, evaluation of postvoid bladder residual is useful even if the woman does not report symptoms of incomplete emptying if the voiding interval is abnormally prolonged. Long labor, peridural analgesia, delivery trauma, prolonged surgical procedures under anesthesia and sequelae of surgical repair (oedema and tissue inflammation) can be the cause, mechanical and/or functional, of diminished perception of the sensation of bladder filling and further secondary damage of the bladder innervation by overdistension.

Overt PPUR (oPPUR) is inability to void spontaneously within 6 h after vaginal delivery or within 6 h after removal of an indwelling bladder catheter after cesarean section, requiring catheterization [28].

Covert PPUR (cPPUR) refers to post void residual volume > 150 mL with voided volume of at least 150 ml [29] with no symptoms of retention.

**1.10:** Rome IV Criteria for Constipation (1.2.3.1): Complaint that bowel movements are

(i) infrequent (< 3/wk);
(ii) need to strain;
(iii) lumpy or hard stool bloating;
(iv) sensation of incomplete evacuation;
(v) sensation of anorectal obstruction or blockage abdominal pain,
(vi) need for manual assistance, in more than one quarter of all defecation.

**1.11:** During pregnancy and in the postpartum period, constipation may be secondary to hormone related slow transit, or other changes associated with anatomical or physiological alteration of the pelvic floor function.

**1.12:** This is a symptom of intraanal and external rectal prolapse, as well as postpartum hemorrhoids. It is reported and measured using the Rockwood Fecal Incontinence Severity Index, and can be equally bothersome to patients as soiling.

## Signs

### Sign

Any abnormality indicative of disease or a health problem, discoverable on examination of the patient; an objective indication of disease [11] or a health problem.

#### Vaginal signs of obstetric pelvic floor trauma

##### Vulvovaginal and perineal obstetric trauma:

Trauma (injury) occurring at the vulva, vagina or perineum at the time of vaginal childbirth [30].

##### Postpartum discharge:

Vaginal clear, bloody or mucus discharge in the postpartum period and up to 12 months after delivery. (NEW).

##### Obstetric cervical trauma:

Trauma occurring to the cervix during the process of cervical dilatation or at the time of childbirth. (NEW).

##### Postpartum vulvovaginal and perineal oedema:

Swelling of the vulvovaginal and perineal area characterized by watery fluid collection. (NEW).

##### Postpartum vulvovaginal hematoma:

Hematoma in the vulva and/or vagina caused during childbirth. (NEW).

##### Vaginal burst trauma:

Multiple generalized multidirectional vaginal tears, usually superficial, that occur during vaginal delivery. (NEW).

##### **Superficial/cutaneous perineal/vaginal trauma** (also known as **first degree tear**):

Laceration of the vaginal epithelium or perineal skin only. On inspection there is cutaneous laceration of the perineal and/or vaginal epithelium. (CHANGED).

##### Postpartum vaginal skin tag:

Excess vaginal skin resulting in skin growth at the introitus diagnosed after vaginal childbirth. (NEW).

##### Vaginal mucosa avulsion/scalp:

Vaginal tear occurring by detachment of the vaginal mucosa from the underlying muscle during vaginal childbirth, resulting in a superficial but wide raw bleeding surface in the vagina. (NEW).

##### Postpartum vaginal granuloma:

Small mass composed of granulation tissue on the surface of a previous wound often secondary to retained suture material or other causes provoking localized foreign body inflammatory reaction following vaginal childbirth and vaginal epithelial injury. (NEW).

#### Postpartum lower urinary tract signs

##### Obstetric bladder injury:

Trauma or injury to the bladder during childbirth. (NEW).

##### Postpartum urinary stress incontinence:

Urinary stress incontinence occurring after childbirth, that was not present before or during pregnancy, observed on clinical examination during cough test, Valsalva or physical exertion. (NEW).

#### Postpartum perineal and anorectal signs

##### **Obstetric perineal muscle trauma** (also known as **second degree**):

Trauma (injury) of the perineal muscles but not the anal sphincter following vaginal childbirth. (CHANGED).

##### **Obstetric anal sphincter trauma (injury)** (also known as **third and fourth degree**):

Disruption of the anal sphincter muscles following vaginal childbirth. (CHANGED).

The different types and classification of obstetric anal sphincter trauma are discussed in the “Diagnoses” Section.

##### Perineal scar:

Cutaneous scar resulting from trauma to the perineum.

##### Deficient perineum:

Reduced perineal body measuring less than 2.5 cm and often associated with introital gaping [31] (NEW).

##### Perineal ridge (PR):

Measurement performed at the hymenal ring that describes the distance in centimeters from the midline of the mid-hymenal ring to the commencement of the posterior vaginal skin on the vaginal side of the PR as distinct from the perineal side (perineal body) [31] (NEW).

##### Cloacal-like defect:

A spectrum of tissue loss from the perineal body and rectovaginal septum with variable appearance. This may consist of a common cavity made up of the anterior vagina and posterior anorectal walls or just an extremely thin septum between the anorectum and vagina [12].

#### Postpartum vulval signs

##### Vulval and perineal ecchymosis (bruising):

Vulval and perineal skin discoloration secondary to subcutaneous blood leakage into surrounding tissue from a broken capillary under the skin. (NEW).

##### Vulval gaping, enlarged (widened) genital hiatus:

Non-coaptation of vulva at rest, commonly associated with increased size of genital hiatus [12]. _FN2.1_

##### Obstetric paraurethral trauma:

Injury of the vaginal epithelium around the urethral meatus at the time of vaginal childbirth. (NEW).

##### Obstetric paraclitoral/clitoral trauma:

Injury close to or of the clitoris at the time of vaginal childbirth. It may or may not involve clitoral tissues. (NEW).

#### Levator ani muscle trauma

##### Levator avulsion:

The detachment of the levator ani muscle (LAM) from its insertion on the inferior pubic ramus [8, 14]. It can be ascertained by per-vaginam palpation, especially during pelvic floor muscles contraction and can be:

- partial
- complete [32]
- unilateral
- bilateral _FN2.2_

#### Obstetric pelvic floor nerve trauma

##### Obstetric neuropathy:

Decreased or absent sensation or tone during examination of the vagina, vulva or perineal structures, following childbirth.

##### Obstetric pudendal nerve injury:

Decreased or absent sensation or tone during examination, in the distribution of the pudendal nerve, secondary to injury to the pudendal nerve or its branches during vaginal childbirth. (NEW).

#### Obstetric pelvic floor musculoskeletal/connective tissue trauma

##### Pelvic organ prolapse (sign):

The descent of one or more of the anterior vaginal wall, posterior vaginal wall, the uterus (cervix) or the apex of the vagina, or the perineum (perineal descent). The presence of any such sign should be correlated with relevant POP symptoms i.e. patient report of symptoms with maximal prolapse. More commonly, this correlation would occur with descent to the level of the hymen or beyond [8, 14, 33–36]. _FN2.3_

#### Bone trauma/effects of pregnancy and childbirth on bones and joints

##### Postpartum symphysis pubis diastasis:

Separation of the symphysis pubis, without fracture, which allows excess lateral or anterior movement of the symphysis pubis and can result in symphysis pubis dysfunction during the postpartum period and up to 12 months after delivery. (NEW).

##### Postpartum coccygeal dislocation/fracture:

The coccyx slips anteriorly or posteriorly with respect to the sacrum during labor and/or postpartum period and up to 12 months after delivery (NEW).

#### Obstetric fistula

Is an abnormal communication from the urinary (bladder, ureter and/or urethral lumen) and/or anorectal tract to the vagina or perineal area respectively characterized by the observation of urine leakage through channels other than the urethral meatus (extra-urethral incontinence) [7] and of feces leakage through channels other than the anal canal observed during postpartum period and up to 12 months after delivery. (NEW). _FN2.4_

##### Extra-urethral incontinence:

Observation of urine leakage through channels other than the urethral meatus, for example, fistula. The fistula may be described anatomically from one structure to another. Below are anatomical descriptions of pelvic floor fistula (PFF). The PFF defects may occur between 2 or more structures [10]. _FN2.5_

##### Lower urinary tract pelvic floor fistula (PFF) signs:

**Fig. 1:**
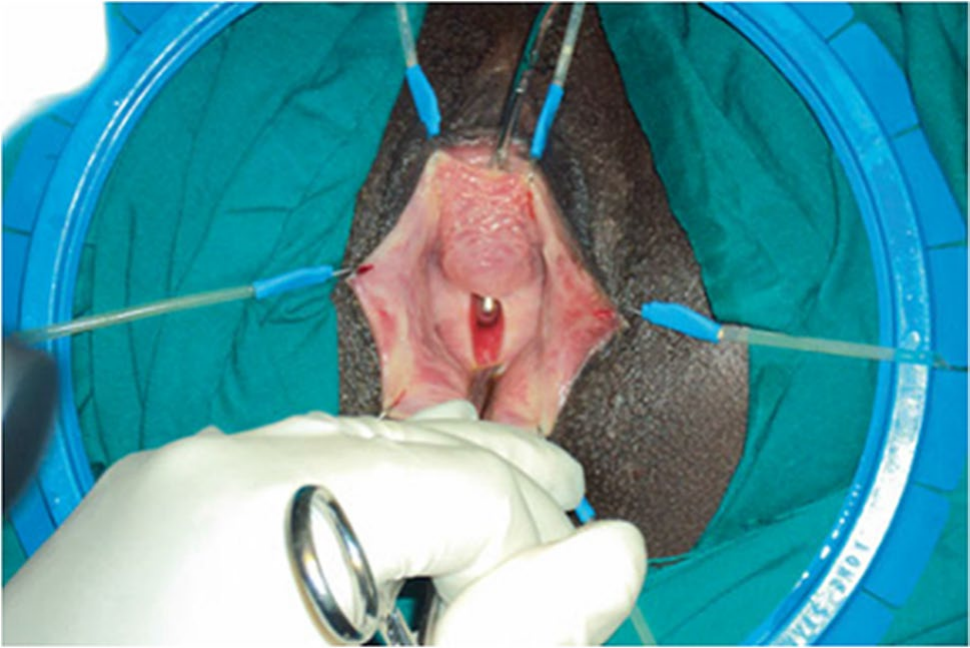
Obstetric vesico-vaginal fistula during repair [37]. *Source:* Reprinted by permission from Springer Nature

**Fig. 2:**
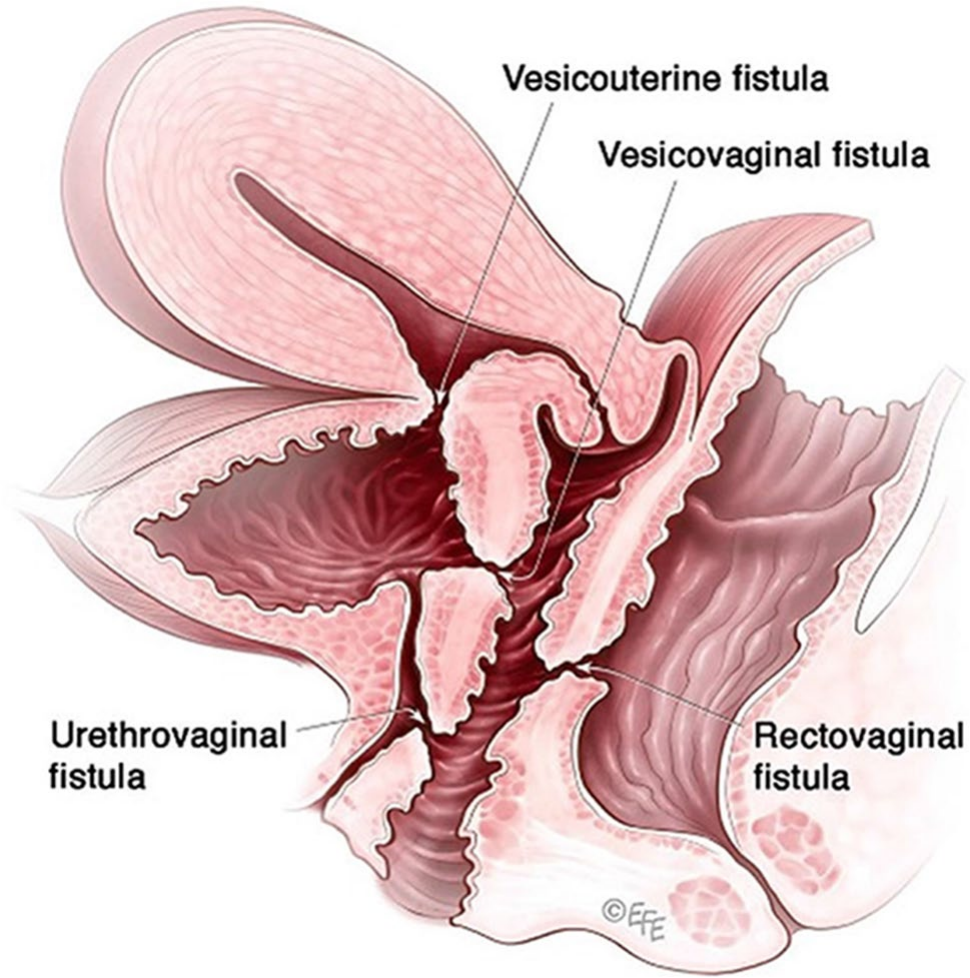
Pelvic floor fistula anatomy © Levent Efe [10]

Observation of a defect between the lower urinary tract structure (urethra, bladder) and vagina (vesicovaginal fistula, Fig. 1) and/or uterus (and/or cervix) that may occur across a spectrum of tissue loss (Fig. 2), with variable appearance and with or without observation of a.

- probe passing through the lumen of the two or more affected structures [10]
- a dyed irrigant fluid passing per defect at the time of retrograde fill test of the bladder through a bladder catheter (positive blue test) [10]
- retrograde blue test after filling to the urethral lumen by a Trattner catheter without filling the bladder [10]. FN2.6

##### Upper urinary tract pelvic floor fistula (PFF) signs:

Observation of a defect between the ureter(s) and vagina and/or uterus (and/or cervix) that may occur across a spectrum of tissue loss, with variable appearance and with or without observation of.

- a probe passing through the lumen of the two or more affected structures [10].
- urine pooling in the posterior vaginal fornix directly or passing through the cervix.
- urine pooling in the posterior vaginal fornix at the time of retrograde dyed irrigation fill test of the bladder through a bladder catheter (negative dye test, positive urine) [10].
- urine passing per cervical os; with or without pooling in the posterior vaginal fornix at the time of retrograde dyed irrigant fill test of the bladder through a bladder catheter (negative blue test, positive clear urine) [10]. _FN2.6_

##### Anorecto-vaginal fistula signs:

Excoriation dermatitis at level of inner thighs, external genitalia, perineum or vagina with or without skin rashes, crusting or scabbing, soiling, discharge, scars, sinus, deformities, hematoma [10].

##### Recto-vaginal fistula signs:

Abnormal connection between the rectum to the vagina with or without observation of vaginal flatus/feces. With or without the observation of:

- anorectal fluid per vagina [10],
- probe or examination finger passing per vagina through anus or per anus through vagina [10],
- anorectal tract fluid per vagina, or with bubbles passing through the abnormal connection through vaginal irrigant fluid after retrograde injection of air per rectum [10]. _FN2.6_

(NEW)

### Clinical Examination

Clinical examination immediately after childbirth or in the postpartum period is guided by parameters of labor, mode of delivery and associated/suspected injuries. Often, acute interventions including emergency measures to arrest postpartum hemorrhage or surgical repair of complex trauma require a thorough clinical examination and accurate diagnosis as well as optimization of analgesia, adequate exposure (positioning, lighting), co-operation and consent of the patient and availability of equipment and assistance. _FN2.7_

#### Postpartum digital vaginal-anorectal examination

An examination in which a doctor or midwife inserts a lubricated, gloved finger into the rectum and a thumb in the vagina. The examiner observes to identify injured structures and palpates the sphincter from 9-o’clock to 3 o’clock to detect lacerations or other injuries to the vaginal or rectal epithelium, perineal muscles and anal sphincter muscles. Anal sphincter tone may be altered by the effects of regional anesthesia. Following a repair, an anorectal exam is performed to detect palpable sutures that have been unintentionally perforated the anorectal epithelium. (NEW).

#### Vaginal examination

Examination for vaginal length and mobility, presence of scarring and/or pain, and estrogenization. The location of any vaginal pain should be noted [6]. _FN2.8_

#### Examination for levator defects/trauma

Per-vaginam palpation for levator injury/defect/ “avulsion” [8, 14].

#### Postpartum rectal examination

Examination of the rectum in the immediate postpartum period and up to 12 months after delivery. The gloved finger should be placed in the center of the anus with the finger parallel to the skin of the perineum in the midline. The finger should then be pressed gently into the anal canal but at the same time pressed backwards against the skin of the posterior wall of the anal canal and underlying sling of the puborectalis muscle. This overcomes most of the tone of anal sphincter and allows the finger to straighten and slip into the rectum [9, 18] (NEW). _FN2.9_

#### Postpartum anorectal examination

A comprehensive anorectal examination undertaken immediately after birth or during postpartum and up to 12 months after delivery. The patient lies in the left lateral position with hips flexed and ankles away from the examiner. Dorsal lithotomy position is also commonly used. An anorectal examination is essential to exclude/confirm obstetric anal sphincter or anorectal injury [9, 18] (NEW).

#### Postpartum perianal and perineal examination

Examination of the perineal and perianal area is conducted around introital-vulvar area, perineum and anus.

Exposure of the vaginal introitus is performed by gently parting the labia majora and minora with inspection and palpation of the external genitalia, the bulbocavernosus and transverse perineal muscles and the vulvar and vaginal epithelium (NEW). _FN2.10_

##### Perianal sensation/reflex:

In patients with possible neurogenic pelvic floor dysfunction there should be particular note of those neurological signs related to S2-4 but these should be complimented by a more general neurological examination as indicated. Specific to anorectal dysfunction, assessment of anal reflex, and perianal sensation should be performed [9, 18].

##### Sacral reflex testing:

This measures the reflex arc of the spinal cord and the sacral plexus including the pudendal nerve. Bulbocavernosus and anal reflex are the most commonly evaluated.

**Footnotes for Section** **Signs**

**2.1:** An enlarged hiatus has long been considered both a possible cause and an effect of pelvic organ prolapse [38]. A large epidemiological study [39] conducted for 10 years on a sample of 1198 women, although not clarifying the causality or not, seems to correlate a larger hiatus and its faster increase with the development of prolapse. A genital hiatus larger than 3.16 cm increasing in the size at a mean rate of more than 0.56 cm per 5-year period could identify women at highest risk for developing prolapse.

**2.2**: Levator avulsion is a discontinuity of the levator muscle at its attachment to the inferior pubic ramus. Discontinuity may represent a partial tear, full tear, or thinning. Test for levator injury/avulsion: palpation of levator tissue, by placing finger(s) between the side of the urethra and the edge of the muscle measured on each side. The test is performed at rest and confirmed by asking the patient to contract and feeling for the edge of the contractile tissue of the levator muscle. RATING: (i) Absent: Palpable pelvic floor muscle contraction next to the urethra on the inferior pubic ramus; (ii) Present: A distance of > 3.5 finger widths between the two sides of puborectalis muscle insertion on pelvic floor muscle contraction. Rate number of finger widths palpable in the gap. Several rating scales exist. Under < 3.5 cm may represent a partial avulsion, however, digital palpation cannot reliably determine this distance of discontinuity [40].

**2.3:** For a more detailed definition, see IUGA/ICS joint report on the terminology for female pelvic organ prolapse [8]

**2.4:** Obstetric fistulas is a rare condition and is usually secondary to mechanical obstetric pelvic floor trauma provoked by a prolonged or obstructed labor in women who do not have access to health care facilities.

**2.5:** Basic categories of Pelvic Floor Fistula are classified defining each in relation to the hollow organ system component involved in the fistula defect (Fig. 2). These are localizing/descriptive terms and not a classification system as such. The following acronyms will be used: F (fistula); V (bladder/vesico); U (urethra); Va (vaginal); Vt (vaginal vault); Ut (uterine); Cx (cervical); Ur (ureteric); R (rectal); Co (colon); Pe (perineal); AC (ano-cutaneous).

**Fig. 3:**
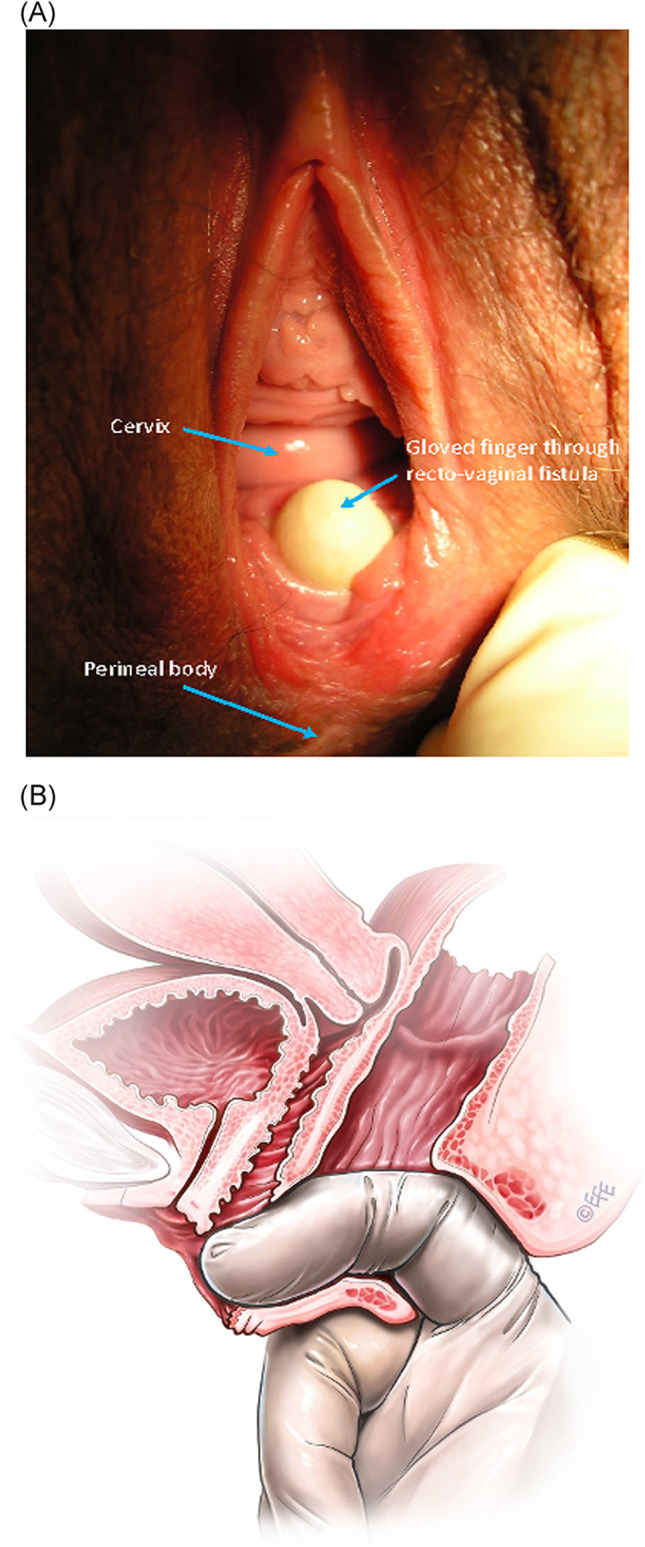
Rectovaginal fistula [10]. Figure 3A: Recto-vaginal fistula, low in the vagina, just proximal to the anus © J Goh; 3B: © Levent Efe

- UVaF: Abnormal connection between the urethra and the vagina [10].
- Vesico-vaginal fistula (VVaF): Abnormal connection between the bladder and the vagina [10].
- Vesico-uterine fistula (VUtF): Abnormal connection between the bladder and the uterus [10].
- Uretero-vaginal fistula (UrVaF): Abnormal connection between the ureter and the vagina [10].
- (Colo)-recto-vaginal fistula (RVaF): Abnormal connection between the rectum (colon) and the vagina [10] (Fig. 3).
- (Colo)-rectal to urinary tract: Any abnormal connection between the rectum (colon) and any part of the urinary tract, without vaginal involvement [10]
- Partial UVaF: Urethral structure is evident, with a demonstrable fistula defect (Fig. 2) [10].
- Total UVaF: Urethral structure is not evident (Fig. 3) [10].
- Circumferential fistula (genito-urinary): An entire segment (anterior, posterior, lateral urethra) from the anterior vaginal wall to the posterior aspect of the pubic symphysis is absent and destroyed. 23,24 The circumferential fistula almost always involves the urethra and the fistula totally separates the proximal urethra/bladder from the distal portion. Bladder involvement with a circumferential fistula is common [10].
- VVaF: Fistula affecting anterior vaginal wall and posterior bladder wall with or without involvement of the ureteric orifices
- Circumferential fistula (genito-urinary): It almost always involves the urethra [10].
- Vesico-vaginal vault fistula (VVtF): VVaF located at vaginal vault (cuff) following hysterectomy [10].
- Vesico-cervical fistula (VCxF): Abnormal connection between the bladder and the cervix. May occur after cesarean section, procedures to the cervix, supra-cervical hysterectomy [10].
- VUtF: Abnormal connection between the bladder and the body of the uterus [10].
- UrVaF: Abnormal connection between the ureter and the vagina [10].
- UrVaF may be congenital (ectopic ureter) [10] or
- Acquired (e.g., following surgery or obstructed labor) [10]
- Uretero-vesical-vaginal fistula (UrVVaF): Fistula involving the ureter(s), bladder, and vagina. This may be seen with a large obstetric fistula and the ureter is outside the VVaF [10].
- Uretero-uterine (cervical) fistula (UrUtF/UrCxF): Abnormal connection between the ureter and the uterus (cervix). Predominantly post-cesarean or postsupracervical hysterectomy [10].
- Fourth-degree tears: Obstetric anal sphincter injury with disruption of the perineal body, connecting the vagina to the anorectum. The internal and external anal sphincters are disrupted [10].
- Acute fourth degree tear—Occurs at time of childbirth or other trauma [10].
- Chronic fourth degree tear—Unrepaired or dehiscence following repair at time of childbirth or other trauma, resulting in an absent perineal body with a total perineal defect [10].
- RVaF: Abnormal connection between the rectum and the vagina [10].
- Non-circumferential RVaF: Involves the posterior vaginal wall and anterior rectum [10].
- Circumferential RVaF: Involves an entire segment of the rectum, involving the posterior vaginal wall, anterior and posterior rectum. The proximal rectal part of the fistula is completely separated from the distal portion [10].
- Rectal/vaginal/perineal fistula (RVaPeF): An abnormal communication from the anorectum to the vagina or perineal area [10].
- Recto-uterine/cervical fistula (RUtF/RCxF): An abnormal connection from the rectum to the uterus or cervix [10].
- Fistula in ano (FIA)/ano-cutaneous fistula (ACF): An abnormal connection between the anal canal epithelium and the skin epithelium.
- Colo-vesical fistula (CoVF): Abnormal connection between the rectum (colon) and the bladder [10].
- Recto (colo)-ureteric fistula (RUrF/CoUrF): Abnormal connection between the rectum (colon) and the ureter [10].

**2.6:** For a more detailed definition see ICS report on the terminology for female pelvic organ fistulas [10].

**2.7:** Training on postpartum management is an essential part of obstetric and gynecological training curriculum [41]. In some countries, childbirth assistance is carried out by doctors who are not specialized in gynecology and few countries provide a specific training in urogynecology during a general obstetrics and gynecology residency [42, 43].

**2.8:** A full vaginal examination should be performed to examine the perineum and vaginal tissues and evaluate the extent of any vaginal tear. Although parting the labia and vaginal walls by the fingers is usually adequate, deep tears may necessitate the use of a Kristeller, Kallmorgen or Sims Vaginal Specula. Evaluation for cervical tear with the sponge-holding forceps gently grasping the cervix to systematically inspect for any disruption is important. A digital rectal examination should be performed to evaluate the anal sphincter complex and to exclude buttonhole tears. The woman can be asked to contract her anal sphincter around the examiners finger.

**2.9: Postpartum rectal examination allows assessment of:**

- Resting anal tone, voluntary squeeze of the anal sphincter as well as the levator muscles, sustained squeeze over 5 s and involuntary contraction elicited during a cough. Immediately after delivery, especially in cases with epidural analgesia, clinicians may not be able to adequately assess the integrity of the sphincter and its tone, which may only to be determined through direct visual examination and its palpation.
- Obvious hemorrhoids can be palpated but grade II and grade III hemorrhoids are better assessed by proctoscopy. Painful examination may be associated with fistula in ano, fissure in ano, infection or pilonidal abscess.
- Palpable anal sphincter gap. An assessment can be made of a palpable anal sphincter gap to assess if there has been recent or previous obstetric or surgical damage. The perineal body can be assessed for deficiency.
- Rectal contents. The contents of the rectum can be assessed. The feces may be hard or soft, the rectum may be empty or collapsed and sometimes ballooned out. This allows assessment of fecal impaction.
- Confirmation of presence of rectocele, enterocele, or perineocele. Use of POP-Q for staging of prolapse.
- Bidigital examination is defined as a concurrent digital examination of the vagina and rectum and may be carried out with the patient supine in a gynecological examining position. The index finger is inserted in the vagina and the middle finger in the rectum, in order to identify fascial rectovaginal defects, exclude a rectovaginal perforation (buttonhole injury) and differentiate a rectocele from an enterocele, during an abdominal straining manoeuvre [9, 18].

**2.10 Perianal and perineal examination aim to assess:**

(i) Perineal body and superficial perineal musculature integrity, consistence and lacerations
(ii) Excoriation: perianal excoriation, skin rashes [9, 18]
(iii) Soiling: perianal fecal soiling or vaginal fecal soiling [9, 18]
(iv) Discharge: perianal or vaginal bloody or mucus discharge [9, 18]
(v) Gaping anus: non-coaptation of anal mucosa at rest [9, 18]
(vi) Scars, sinuses, deformities, condylomata, papillomata, hematoma
(vii) “Dovetail” sign: where the anterior perianal folds are absent, indicating a defect in the external anal sphincter [23]
(viii) Others described individually: anal fissure, hemorrhoids, anorectal prolapse, fistula-in-ano, recto-vaginal fistula, anorectal/ vaginal/perineal fistula [9, 18] (Fig. 3)

## Investigations

### Assessment of possible impact of obstetric trauma on voiding function

Labor, regional anesthesia and delivery and specifically obstetric pelvic floor trauma can have a negative impact on voiding function, screening for which involves a bladder postvoid residual volume measurement and ideally uroflowmetry. Voiding cystometry may clarify the cause of any voiding dysfunction.

However, invasive urodynamic investigations in the postpartum period are usually delayed and non-invasive assessment (post void residual measurement) is the common investigation of choice for first line assessment of voiding symptoms. (NEW).

#### Postpartum urodynamic investigations

Measurement of physiological parameters relevant to the function of the lower urinary tract [7].

These usually take place in a special clinical room and involve post void residual urine volume (PVR) measurement after a spontaneous micturition flow. Uroflowmetry, filling cystometry with (artificial) bladder filling with a specified liquid (ICS recommends physiological saline solution or X-ray contrast if video studies) at a specified rate and pressure-flow studies are unnecessary in the most, [44] if not, in almost all cases during postpartum period and up 12 months after delivery. _FN3.1_ (NEW).

##### Postpartum post void residual (PPVR) measurement:

Volume of urine left in the bladder at the completion of voiding in the early post-partum period (up to 4 weeks), that may be measured by catheter or ultrasound [6, 7, 15, 45] (NEW). _FN3.2_

##### Postpartum free (no catheter) uroflowmetry:

**Fig. 4:**
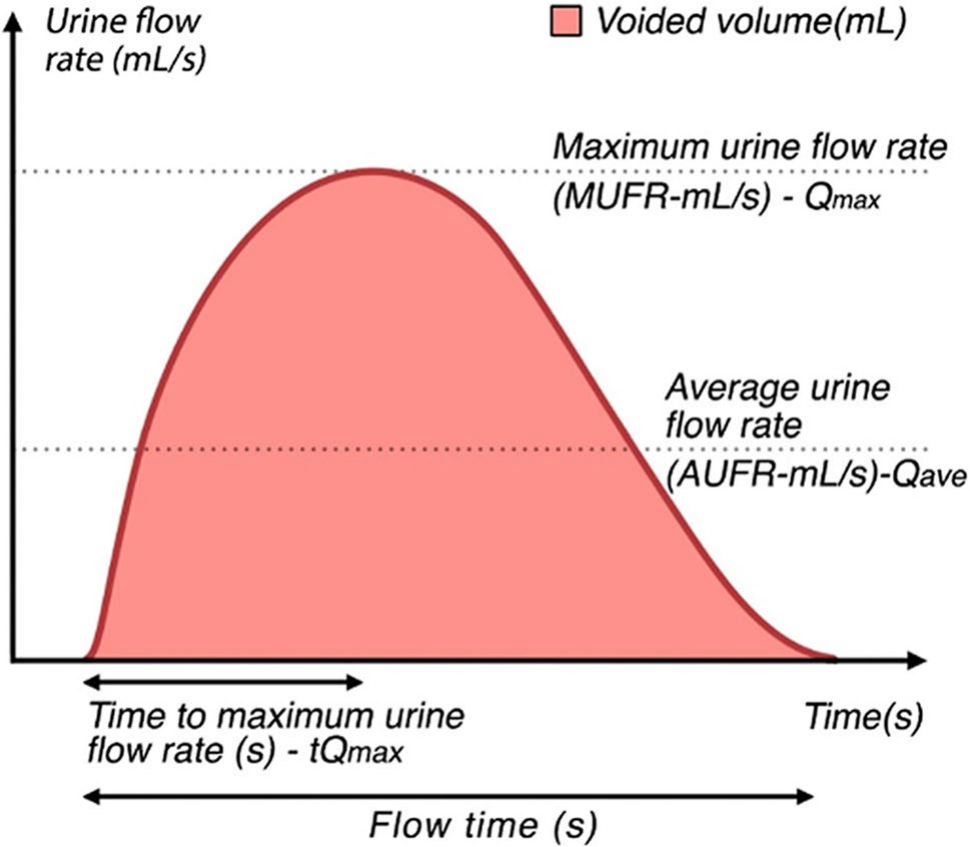
Uroflowmetry A schematic representation of urine flow over time and parameters of uroflowmetry [10]. (Republished with permission of John Wiley & Sons—Books, permission conveyed through Copyright Clearance Center, Inc)

Measurement of urine flow rates during micturition. A test that measures the flow rate of the external urinary stream as voided volume per unit time in milliliters per second (mL/s) [46] (Fig. 4). _FN3.3_

### Assessment of possible impact of obstetric trauma on defecatory and anorectal function

#### Anorectal manometry

Assessment of resting, squeeze pressures as measured with air or water charged or solid state pressure manometer. As normal values can differ substantially between laboratories according to the style of catheter used, each unit is encouraged to generate its own normal data [9, 18].

### Imaging

#### Ultrasound 3D and 4D

The potential of 3D ultrasound in urogynecology and female urology is currently being researched with validated applications likely to be included in future updates of this report and/or separate ultrasound reports. Applications with the most current research include: (i) major morphological abnormalities such as levator defects, (ii) excessive distensibility of the puborectalis muscle and levator hiatus (“ballooning”) and anal sphincter integrity. The additional diagnostic potential of 4D (i.e., the addition of movement) ultrasound awaits clarification by further research [6].

#### Endoanal ultrasound (EAUS) or anal endosonography (AES)

**Fig. 5:**
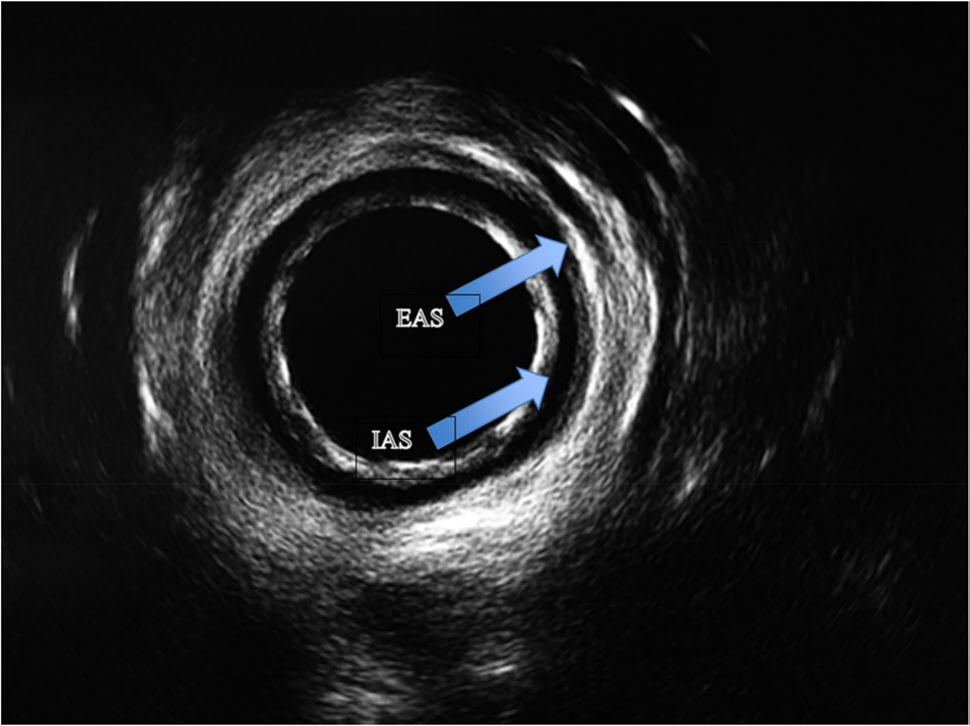
Endoanal ultrasound images showing normal appearance of the internal (IAS) and external (EAS) anal sphincters [47]. *Source:* Reprinted by permission from Springer Nature

Ultrasound of the anal canal performed with a pole-like ultrasound probe placed in the anal canal giving a 360 degree image of the anal canal (Figure). It is usually performed with the patient placed in the lithotomy, prone position or sometime left lateral. Two dimensional AES; three dimensional AES—three-dimensional reconstruction of the anal canal is performed using either axial or sagittal images [7] (Fig. 5). _FN3.4_

#### Ultrasound imaging modalities[6]

The practice parameters for terminology and the practice parameters for performance of urogynecological examinations were established by the American Institute of Ultrasound in Medicine (AIUM) and IUGA in collaboration with the AUGS, AUA, ACR, and SRU in 2019 [48].

##### Perineal:

Curved array probe applied to the perineum. This term incorporates transperineal and translabial ultrasound.

#####  Introital:

Sector probe applied to the vaginal introitus.

##### Transvaginal (T-V):

Intravaginal curvilinear, linear array or sector scanning. An endocavity transducer is inserted into the vagina.

##### Transabdominal (T-A):

Curvilinear scanning applied to the abdomen.

##### Ultrasound 3D imaging of levator ani trauma:

The presence of levator ani trauma has been postulated to be associated with an increased risk of pelvic organ prolapse. This can be evaluated using a tomographic ultrasound imaging assessment of the levator ani muscles [8].

#### Transperineal ultrasound imaging criteria for levator ani avulsion are defined as:

##### Levator avulsion complete:

**Fig. 6:**
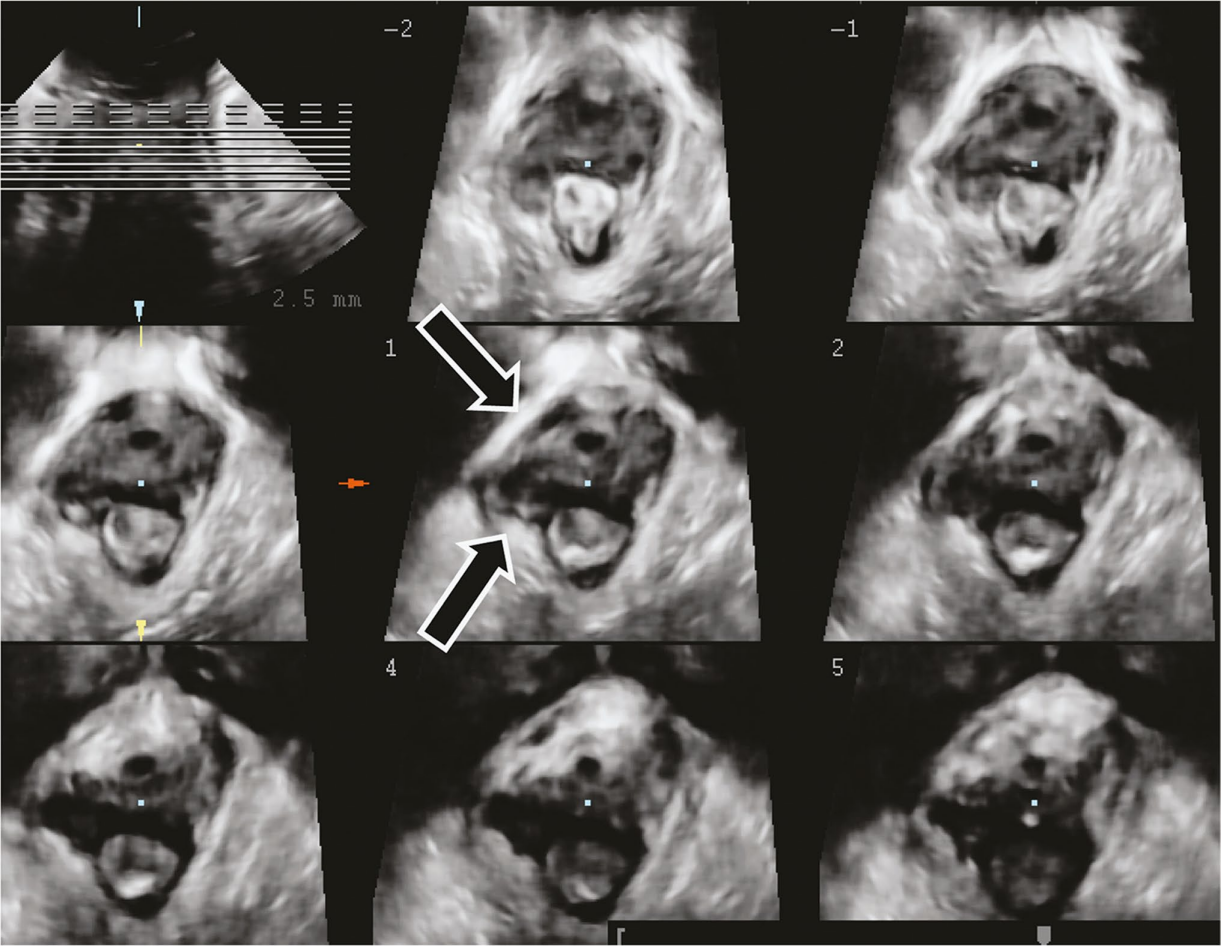
Ultrasound image of the pelvic floor in a woman following forceps vaginal delivery, showing avulsion of the levator plate from the right pubic ramus. Courtesy of Professor HP Dietz [49]. *Source:* Reproduced with permission of the Licensor through PLSclear

A defect in 3 central slices as identified on transperineal imaging (Fig. 6) (NEW).

##### Levator avulsion partial:

Defect in 1 − 2 / 3 central slices as identified on transperineal imaging (NEW).

##### Tomographic ultrasound imaging (TUI):

**Fig. 7:**
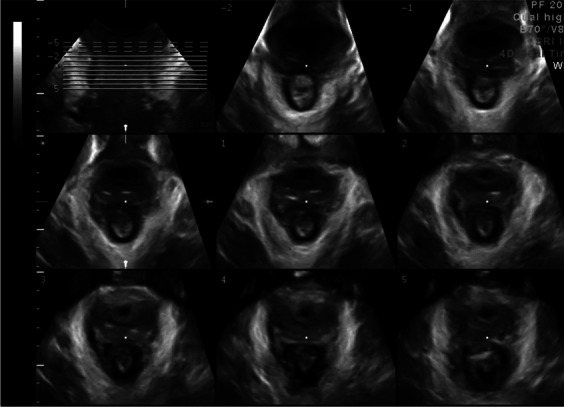
Tomographic ultrasound imaging assessment of the levator ani muscles Intact LAM [8, 14]. (Republished with permission of John Wiley & Sons—Books, permission conveyed through Copyright Clearance Center, Inc)

Can be used for assessment for puborectalis avulsion should be performed on volumes obtained during a pelvic floor muscle contraction at 2.5-mm slice intervals, from 5 mm below to 12.5 mm above the plane of minimal hiatal dimensions [50] (Fig. 7).

##### 3D ultrasound imaging of ballooning of the genital hiatus:

**Fig. 8:**
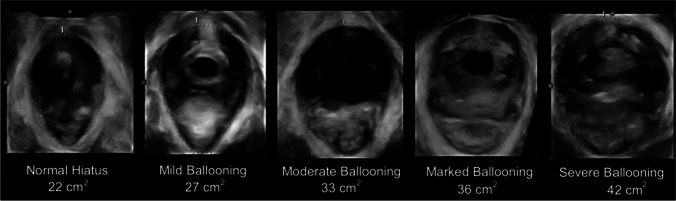
Ballooning of the genital hiatus on straining manoeuvre—levator defect [8, 14]. (Republished with permission of John Wiley & Sons—Books, permission conveyed through Copyright Clearance Center, Inc)

The presence of ballooning of the genital hiatus (= excessive distensibility of the levator hiatus) on straining manoeuvre has also been associated to the severity of urogenital prolapse. An area of more than 25 cm ^2^, 30 cm ^2^, 35 cm ^2^ and 40 cm ^2^ has been defined as mild, moderate, marked and severe ballooning respectively (Fig. 8) [8, 14, 51].

##### Clinical applications of pelvic floor Ultrasound:

- Bladder neck descent/mobility. The position of the bladder neck at rest and on straining.
- Urethral funneling: i.e., opening of the proximal third of the urethra during coughing or on straining. _FN3.5_
- Post void residual: Several formulas have been described in the literature to measure the bladder volume by ultrasound [7].
- Rectal intussusception [9]
- EAS, IAS injury
- Degree of anal mucosa coaptation [52]

##### Postpartum ultrasound imaging modalities limitations and benefits: endovaginal, transanal, and translabial/transperineal:

- Endovaginal ultrasound imaging may inadvertently compress tissues thus distorting the anatomy.
- Transanal ultrasound approach requires an expensive and dedicated transducer, and it is a more uncomfortable and embarrassing test for the woman. Its most common clinical indication is the assessment of sphincter integrity following obstetric trauma. It is the reference standard for imaging of the anal sphincters and diagnosis of sphincter defects and correlates with symptoms and histological diagnosis [53].
- Translabial/transperineal approach overcomes the limitations of endovaginal and transrectal techniques providing minimal pressure on local structures and it is least likely to alter surrounding anatomy. There is ongoing research validating this against the transanal approach for diagnosis of sphincter integrity and correlation with symptoms. (NEW).

##### Postpartum imaging evaluations:

The following pelvic floor abnormalities can be evaluated following childbirth:

- trauma (injury/damage) of the levator ani muscle (LAM)
- excessive distensibility of the puborectalis muscle and levator hiatus (“ballooning”)
- pathologies of the anterior vaginal compartment like urethral diverticula.
- anal sphincter integrity
- hematomas
- voiding symptoms
- defecatory symptoms
- excessive pain or pressure (NEW).

#### Magnetic resonance imaging (MRI) of the pelvic floor

MRI allows the detection of ligamentous and muscular pelvic floor structures in fine detail. Although it does not use ionizing radiation, it is a high cost technique, may not be suitable for all patients and, in most centers, is not a dynamic study. _FN3.6_

##### Static MRI:

relies on static sequences and high spatial resolution images, to delineate the passive and active elements of the pelvic organ support system. Most commonly, images are acquired in axial, sagittal and coronal planes. MRI has been proposed to be a useful method for diagnosing and staging POP. Several lines and levels of reference have been described in the literature. The most commonly used ones are either a line drawn from the inferior margin of the symphysis pubis to the last coccygeal joint (pubococcygeal line—PCL), in the sagittal plane, noted as midpubic line (MPL). Other applications of MRI are the assessment of the LAM morphology (size, thickness volume) and detection of LAM injuries/defects/(“avulsion”).

#### Levator trauma MRI based diagnosis

##### Levator avulsion unilateral:

The disruption of the levator ani on only one side visualized on MRI. Levator avulsion refers to thediscontinuity of the levator muscle at its attachment to the inferior pubic ramus. (NEW).

##### Levator avulsion bilateral:

The disruption of the levator ani on both sides visualized on MRI. Levator avulsion refers to the discontinuity of the levator muscle at its attachment to the inferior pubic ramus [54] (NEW).

##### Dynamic MRI:

Is a technique that enables imaging of the mobility of the pelvic floor structures on straining. (NEW).

#### Computed tomography (CT) of the pelvic floor

Computed tomography (CT) may offer an accurate visualization of the pelvic floor soft and bony structures by reconstruction of axial images using 1 mm thick slices without gaps thus increasing the diagnostic accuracy of pelvic floor anatomical disorders (i.e. LAM trauma). However, multiplanar spiral CT is not routinely recommended for imaging the pelvic floor, in the postpartum period or during the breastfeeding period, mainly due to irradiation and poor soft tissue contrast. (NEW).

### Electrophysiologic Testing

#### Electromyography (EMG)

Is the recording of electrical potentials generated by the depolarization of muscle fibers. Electromyographic diagnosis is made by evaluating the state of the muscle (muscle pathology) by recording and analyzing the electrical activity generated by the muscle. 1. Intramuscular EMG: insertion of a wire or needle electrode into the muscle to record motor unit action potentials. 2. Surface electromyography: electrodes placed on the skin of the perineum or inside the urethra, vaginal or rectum [55].

#### Pudendal nerve terminal motor latency testing

Is a measurement of time from stimulation of the pudendal nerve to muscular contraction of the bulbocavernosus or external anal sphincter. The St. Mark’s pudendal electrode (Medtronic functional diagnostics A/S) can be used to stimulate the nerve via the rectum or vagina. (NEW).

#### Transvaginal pudendal nerve terminal motor latency testing

The St. Mark’s pudendal electrode (Medtronic functional diagnostics A/S) may be used to measure time from stimulation of the pudendal nerve to muscular contraction of the bulbocavernosus or external anal sphincter (NEW).

#### Transient pudendal nerve terminal motor latency

Increased pudendal nerve terminal motor latency identified postpartum that resolves in a short time interval within 8 weeks [56] (NEW).

### Associated radiological modalities

**Defecography** demonstrates normal anatomy of the anorectum as well as disorders of rectal evacuation. Barium paste is inserted rectally prior to defecation over a translucent commode. Measurement of the anorectal angle is allowed with evidence of the presence, size or emptying of any rectocele. Enterocele, rectal intussusception and mucosal prolapse might be diagnosed as well as a spastic pelvic floor (anismus) [6]. (Unchanged).

### Dye and bubble tests for pelvic floor fistulas

Dye tests may be used to detect small or unusual fistulas (less useful for large or multiple fistulas), such as utero-vaginal or cervico-vaginal fistulas and to differentiate ureteric fistula (clear or yellow urine in vault, “negative dye test with urine in vault”) from bladder fistula (“positive dye test”) or to detect small or distorted anorectal fistula (positive vaginal bubble or rectal dye test) [10].

#### Simple dye test for urinary tract fistulas

The bladder is filled retrograde through a urethral catheter using a dye to change the color of the irrigation fluid, for example, methylene blue or indigo carmine to turn the irrigation fluid blue (Fig. 10). Observation may begin with or without retractor(s) in the vagina, depending on digital and visual exam signs and patient symptoms, or following careful dissection. Blue fluid leakage per genital tract or per anus indicates a bladder or urethral fistula. Lack of blue fluid leakage combined with visualization of extra-meatal clear urine leakage increases suspicion of an upper urinary tract ureteric fistula [10].

#### Triple swab test for urinary tract fistula

Three separate sponge swabs, one above the other, are placed in the upper, middle, and lower vagina. The bladder is then filled with a pigmented irrigant such as diluted methylene blue, and the swabs are removed after 10 min (it can take up to 30 min for urine to come through a tiny tortuous fistula especially if it is in the cervix or uterus). Discoloration of only the lowest swab supports diagnosis of a low urethral fistula or urethral leakage. Diagnosis of a uretero-genital fistula is supported when the uppermost swab is wet but not discolored. A VVaF fistula diagnosis is supported when the upper swabs are wet with blue irrigant. Careful observation for backflow of blue irrigant per meatus must be ongoing to avoid false-positive test reporting [10]. _FN3.7_

Complex of multiple urinary tract fistulas concurrent between the ureter and uterus/cervix and between the bladder and uterus/cervix are often diagnosed by hysterosalpingogram (HSG) or contrast-enhanced MRI [10].

**Footnotes for Section** **Investigations**

**3.1:** During pregnancy significant changes in the anatomy (macro and microscopic) and physiology of the lower urinary tract due to mechanical and hormonal factors that together with the expansion of the uterus and the weight of the foetus, especially in predisposed women, can result in the occurrence of UI [57].

The prevalence of UI goes from 26% of the prepregnant state to 58% in the third trimester of pregnancy even if it is mainly a mild SUI characterized by only droplet losses, less than once a week [58].

The prevalence and incidence of urinary incontinence in pregnancy are always less than 54% and 67% respectively, in the postpartum decreasing respectively below 21% and 45% with the multiparous women reaching values higher than about 10% compared to primiparous in pregnancy and vice versa in the puerperium [59]. More than half of women who experience any type of urinary loss during pregnancy, whether the symptom was present before pregnancy (55%) or who experience it for the first time in pregnancy (60%), consider it a minor problem or ignore it [60]. Lifestyle changes, including pelvic floor muscle training, before and/or during pregnancy and in the postpartum period could be effective (reaching a grade of recommendation A or B) in preventing UI [61] and urodynamic investigations are not necessary before rehabilitation treatment. This, together with lifestyle modification represents the first therapeutic choice in pregnancy and in the postpartum period [62]. In the first six months after childbirth there is a slow recovery of the pre-gravid anatomical-functional conditions even if some of these will never return as before pregnancy [63]. Breastfeeding itself may delay the reestablishment of a regular hormonal cycle. In pregnancy and postpartum, more invasive, pharmacological or surgical interventions should not be carried out [57, 62, 63].

**3.2: Conditions for PVR measurement:** PVR reading is erroneously elevated by delayed measurement due to additional urine production (1–14 mL/min). Ultrasonic techniques (transvaginal, translabial most accurately) allow immediate (within 60 s of micturition) measurement and possible repeat measurement. A short plastic female catheter provides the most effective bladder drainage for PVR measurement by catheterization [8].

Assessment of normality of PVR: Quoted upper limits of normal may reflect the accuracy of measurement. Studies using “immediate” PVR measurement (e.g., transvaginal ultrasound) suggest an upper limit of normal of 30 mL. Studies using urethral catheterization (up to 10 min delay) quote higher upper limits of normal of 50 or 100 mL. An isolated finding of a raised PVR requires confirmation before being considered significant [6].

The accuracy of bladder scanners for measuring PVR in the postpartum period has been questioned, [64, 65] however a number of validation studies have shown they are precise and reliable, and preferred over catheterization [66, 67]. If in doubt in/out catheterization may be performed.

The postvoid residuals (PVRs) of women more than 4 weeks postpartum can generally be regarded as for other women with symptoms of lower urinary tract dysfunction. Normal: zero mls; Small: under 30 mls (ultrasound measurement) or 50 mls (catheter measurement); Moderate: 30 mls to 100 mls; High: Over 100 mls. In the early postpartum period (up to 4 weeks) [68, 69]

**3.3:** For a more detailed definition see ICS report on the terminology for female pelvic organ fistulas [10].

**3.4:** EAUS can be used in the assessment of sphincter integrity following obstetric trauma. Although, clinical assessment remains the most commonly used modality to diagnose perineal trauma in the immediate postpartum period, ultrasound has been also used. Postpartum ultrasound examination can be associated with an improvement in diagnosis of anal sphincter tears [70].

**3.5:** A deep urethral funneling ≥ 50% of the urethra is a sign of SUI or occult SUI [71].

**3.6: Clinical applications of MRI:**

**Fecal incontinence:** Endoanal ultrasound and endoanal magnetic resonance imaging (MRI) have been demonstrated to be comparable in the detection of external sphincter defects. External phased array coil MRI can replace endoluminal MRI with comparable results.

**Levator ani injuries:** Abnormalities of the LAM are identified on MRI as present or absent. Defect severity is further scored in each muscle from 0 (no defect) to 3 (complete loss). A summed score for the two sides (0–6) is assigned and grouped as minor (0–3) or major (4–6).

**Obstructed defecation:** During maximal straining manoeuvre, dynamic MRI may be used to demonstrate: **Rectocele:** measured as the depth of wall protrusion beyond the expected margin of the normal anorectal wall. Based on sagittal MR-sections through mid of pelvis, rectoceles are graded as small (< 2 cm), moderate (from 2 to 4 cm), and large (> 4 cm).

**Rectal intussusception:** The infolding of the rectal mucosa occurring during defecation. Depending on the location, an intrarectal intussusception, limited to the rectum, is distinguished from an intra-anal intussusception extending into the anal canal. The location of the intussusception may be anteriorly, posteriorly, or circumferentially. The intussusception either involves only the mucosa or the full thickness of the rectal wall.

**Enterocele:** Defined as a herniation of the peritoneal sac, which contains omental fat (peritoneocele), small bowel (enterocele) or sigmoid (sigmoidocele), into the rectovaginal or rectovesical space below the PCL. The largest distance between the PCL and the most inferior point of the enterocele is measured with a perpendicular line. Depending on this distance, small (< 3 cm), moderate (3–6 cm), and large (> 6 cm) enteroceles are distinguished.

**Dyssynergic defecation:** Different structural imaging findings can be seen on dynamic pelvic MRI, including prominent impression of the puborectal sling, narrow anal canal, prolonged evacuation, a lack of descent of the pelvic floor and thus a failure to increase the ARA.

The anorectal angle is the angle created by a line drawn through the central axis of the anal canal and a line drawn through either the central axis of the distal rectum or a line drawn parallel to the posterior wall of the distal rectum [72].

In comparison with clinical examination (POP-Q), dynamic MRI has no additional value in the prediction of symptoms with increasing degree of POP.

**Perianal abscesses and fistulas** [9].

**Bladder neck and cervical descent/mobility:**

- Position of bladder neck and cervix at rest and on straining
- Pubo-coccygeal line: A line extending from the inferior border of the pubic symphysis to last coccygeal joint (pubococcygeal line—PCL) Bladder neck or cervical descent > 2 cm below this line with straining indicates weakness of the pelvic floor. If alternative landmarks are used in scientific papers they should be clearly described [73].
- PICS line (Pelvic Inclination Correction System line): this line takes into account the pelvic inclination during straining or Valsalva. The use of this line is recommended for dynamic MRI. For measurements outside the midsagittal plane, the 3D PICS is the most advanced measurement system for any kind of pelvic floor disorders [74, 75].

**3.7:** Other dye and bubble tests for Pelvic Floor Fistulas are:

- **Double dye test for urinary tract fistula**: This includes oral intake of phenazopyridine (pyridium) 200 mg three times a day for one to two days until urine is bright orange, followed by retrograde bladder filling with blue irrigant through a bladder catheter. Diagnosis of a bladder or urethral fistula to the vagina (VVaF, UVaF) is supported if the vaginal swab turns blue. Diagnosis of a ureteric fistula to the vagina is supported if the swab turns orange, combination upper and lower urinary tract fistula to the vagina is supported if the swab turns both blue and orange. Careful observation for backflow of blue irrigant per meatus must be ongoing to avoid false-positive test reporting [10].
- **Trattner double balloon catheter test for urethral fistula:** The Trattner catheter has two balloons, one sits intravesically and the other inflates outside of the meatus to block efflux from the urethra. The irrigant flows out through a lumen that sits between the balloons, isolating fill to the urethra [10].
- **Posterior wall irrigant/fluid per rectum for anorectal tract fistula:** As with bladder dye testing, dye irrigation fluid may be instilled per rectal catheter. If colored irrigant passes per vagina, an anorectal fistula to the genital tract is confirmed [10].
- **Posterior wall “bubble test” for anorectal tract fistula:** With anterior vaginal wall retraction permitting visualization of the posterior vaginal wall, a Foley catheter is inserted into the rectum, the balloon inflated, and held under gentle traction against the anus. Irrigant fluid is placed per vagina. A catheter-tipped, air-filled syringe is inserted into the catheter and slowly decompressed to insert air into the rectum. Vaginal inspection allows visualization of bubbles emanating per vagina through a fistula defect [10].

## Diagnoses (including complications)

This Report highlights the need to base diagnoses for the different types of obstetric pelvic floor trauma on the correlation between a woman’s symptoms, signs and any relevant assessment based on clinical examination and diagnostic investigations.

### Pelvic floor, vaginal and perineal diagnoses

#### Obstetric perineal injury

Injury to perineum occurring at the time of vaginal childbirth [30] (NEW) and usually classified using the RCOG criteria. _FN4.1_

This is further subdivided into:

##### First-degree tear:

Injury to perineal skin and/or vaginal mucosa. (NEW)

##### Second-degree tear:

Injury to perineum involving perineal muscles but not involving the anal sphincter. (NEW).

##### Third-degree tear:

**Fig. 9:**
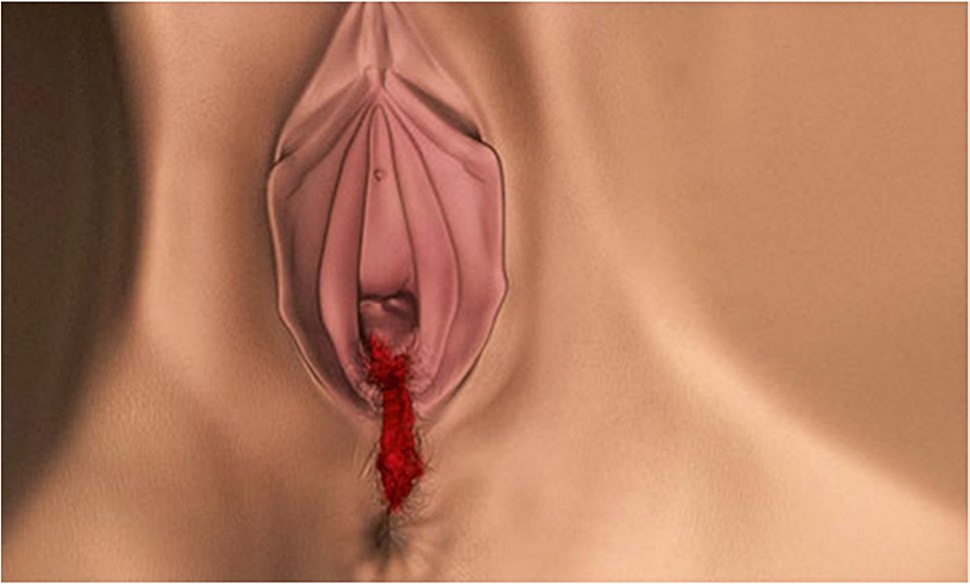
External appearance of a third degree tear [47] *Source:* Reprinted by permission from Springer Nature

Injury to perineum involving the anal sphincter complex (Fig. 9). Tears involving the external anal sphincter can be:

- **Partial EAS tears**
- **Complete EAS tears (NEW)**

##### Grade 3A tear:

< 50% thickness of external anal sphincter torn. (NEW).

##### **Grade 3B tear**:

> 50% thickness of external anal sphincter torn. (NEW).

##### Grade 3C tear:

**Fig. 10:**
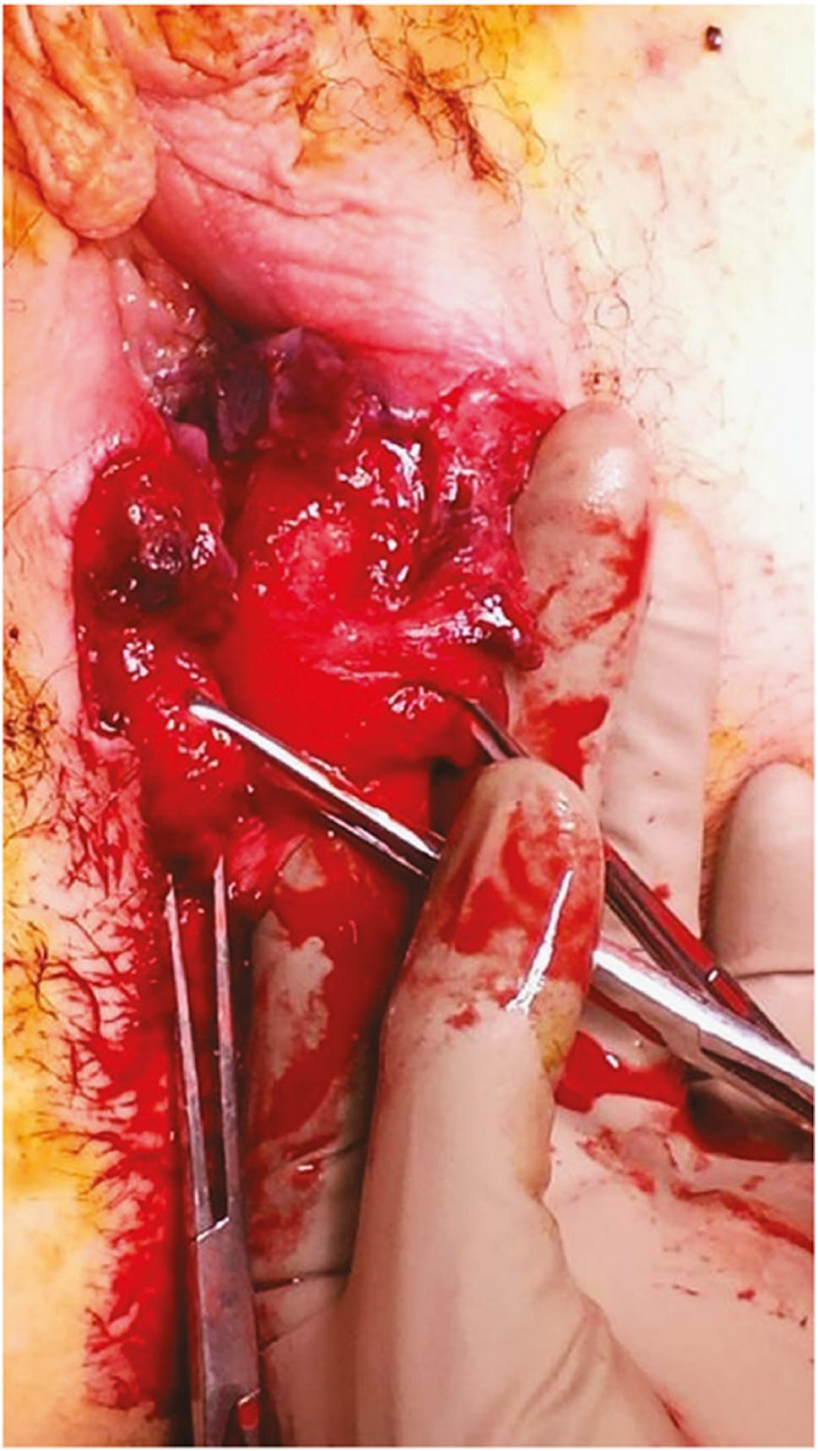
Third-degree perineal tear, with complete division of the internal and external anal sphincters [49]. *Source:* Reproduced with permission of the Licensor through PLSclear

External and internal sphincters torn (Fig. 10). (NEW).

##### Fourth degree tear:

**Fig. 11:**
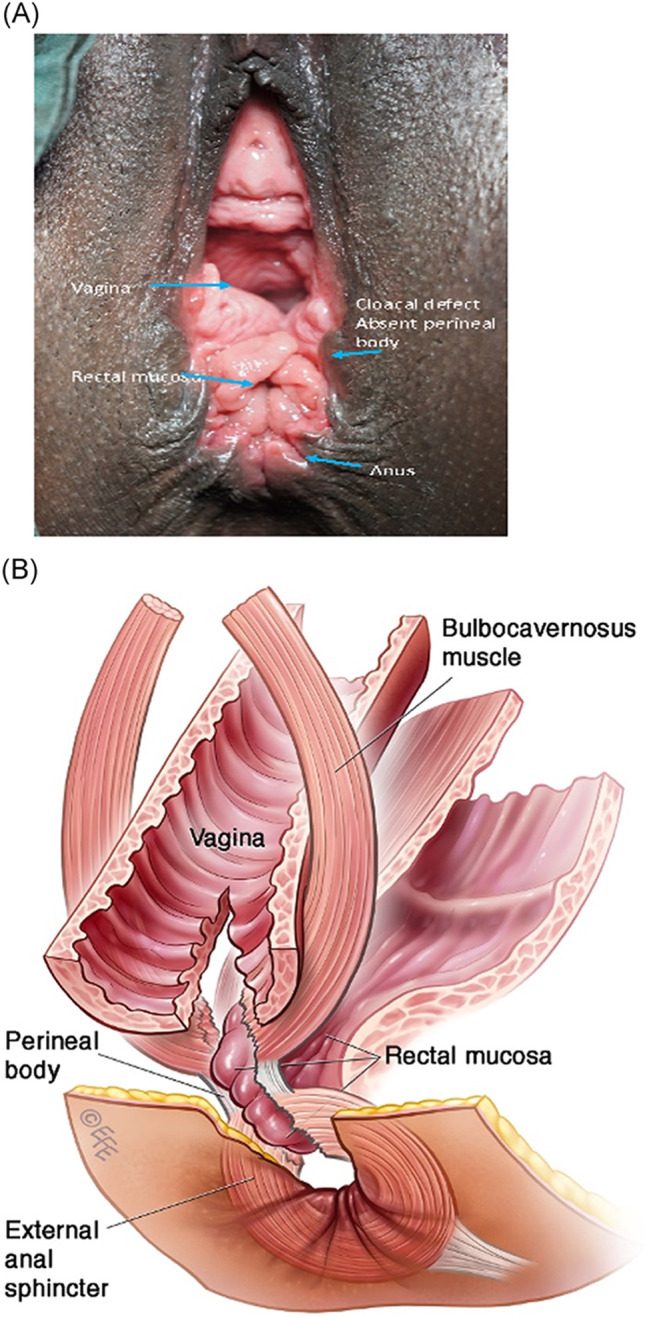
A, B: (A) Fourth degree rectovaginal tear with perineal body disruption. Congenital defects of a similar configuration may also occur. © J Goh. (B) © Levent Efe [10]

**Fig. 12:**
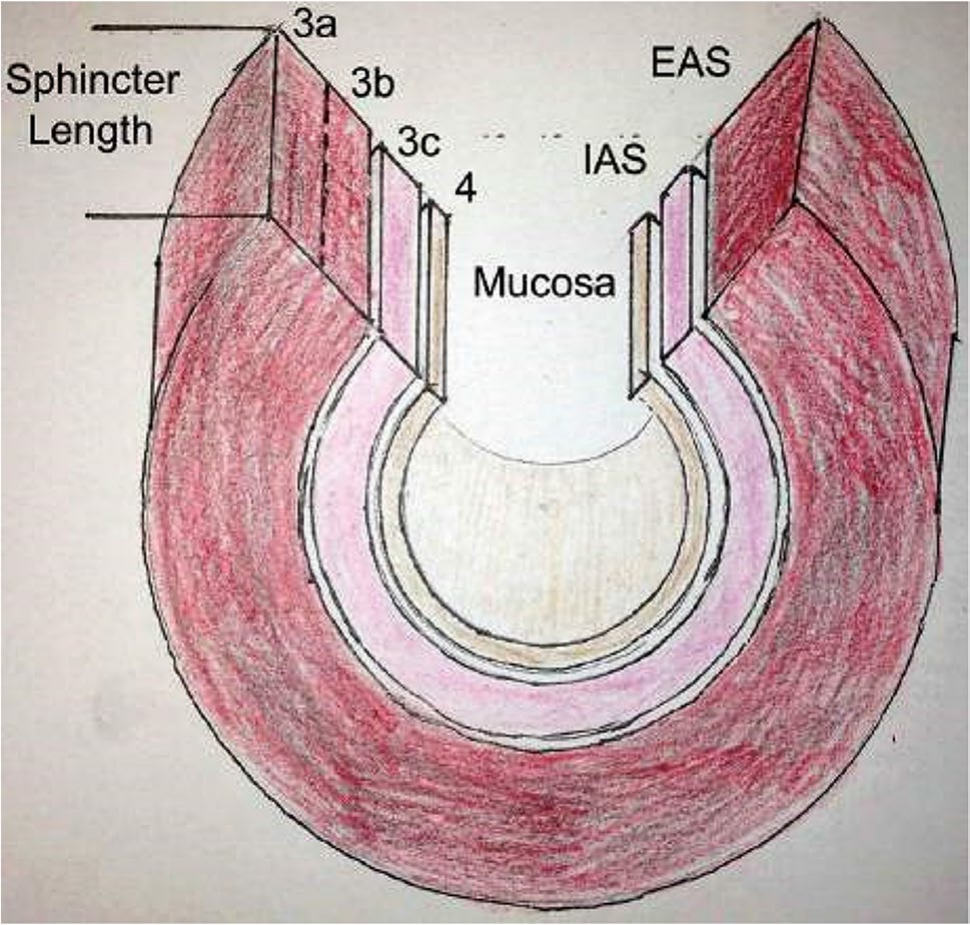
Classification of anal sphincter injuries [76] *Source:* Reproduced with kind permission from publishers

4.1.1.4 : Injury to perineum involving the anal sphincter complex (EAS and IAS) and anorectal mucosa. (Figs. 11 and 12) (NEW).

##### **Obstetric rectovaginal perforation** (also known as “**buttonhole” tear**):

Trauma (injury) of the anal mucosa and the vaginal epithelium without involvement of the anal sphincters. (NEW).

##### **Cloacal defect:**

A confluence of the anus and vagina with no perineum to divide the vagina and anus, leaving one large opening. (NEW).

##### Obstetric fistula (OF):

De novo fistula due to prolonged obstructed labor causing pressure necrosis of soft pelvic tissues between the impacted fetal presenting part and the bony maternal pelvis caused by ischemia and necrosis resulting in an abnormal communication between the urinary/colorectal tract and the vagina or perineal area during the postpartum period and up to 12 months after delivery. (NEW). _FN4.2_

##### Iatrogenic childbirth-related postpartum fistula (ICRF):

Fistula is directly due to inadvertent injury to urinary/colorectal tract during operative delivery (cesarean section/cesarean hysterectomy or instrumental delivery including episiotomy) [10]. (NEW).

##### Mixed obstetric and iatrogenic fistula (MOIF):

Fistula related to operative delivery for prolonged obstructed labor [10]. Tissue integrity already compromised by obstructed labor prior to operative delivery.

##### Obstetric genitourinary fistula:

An abnormal connection between the genital tract and urinary tract [10].

##### Genito-anorectal tract fistula:

An abnormal connection between the genital tract (vagina/uterus/cervix) and the anorectum [10].

##### Fistula in-ano:

An anal fistula is an abnormal connection between the anal canal epithelium (or rarely rectal epithelium) and the skin epithelium. Patients may complain of pain, swelling, intermittent discharge of blood or pus from the fistula and recurrent abscesses formation [9].

##### Anal fissures:

Longitudinal split in the skin of the anal canal, exposing the internal anal sphincter muscle and causing anal spasm. Often associated with pain, a tearing sensation and fresh blood with defecation. Most fissures are found in the mid-line posteriorly and there may be a skin tag associated with them. The second most common gastrointestinal complication after hemorrhoids [9].

##### Hemorrhoids:

Dilated and engorged blood vessels in swollen tissue internally in the anal canal or externally around the anus, that may be characterized​ by bleeding, pain, or itching. Internal hemorrhoids may protrude through the anus [77]. (NEW). _FN4.3_

#### Vulvovaginal complications in the postpartum period

##### Postpartum vaginal fusion (agglutination):

Where the walls of the vagina are stuck together during the postpartum period and up to 12 months after delivery [12]. (NEW).

#### Vaginal narrowing: Decreased vaginal calibre

##### Postpartum scarred vagina:

Self-perception or perception by the partner of a “stiff “ vagina or a foreign body in the vagina in the postpartum period and up to 12 months after delivery. (NEW).

##### Postpartum vulvo-vaginal hyperaesthesia:

Increased vulvo-vaginal sensitivity to touch, pressure, vibration or temperature during the postpartum period and up to 12 months after delivery. (NEW).

##### Postpartum vulvo-vaginal hypoaesthesia:

Reduced vulvo-vaginal sensitivity to touch, pressure, vibration or temperature during the postpartum period and up to 12 months after delivery. (NEW).

##### Postpartum perineal wound infection:

**Fig. 13:**
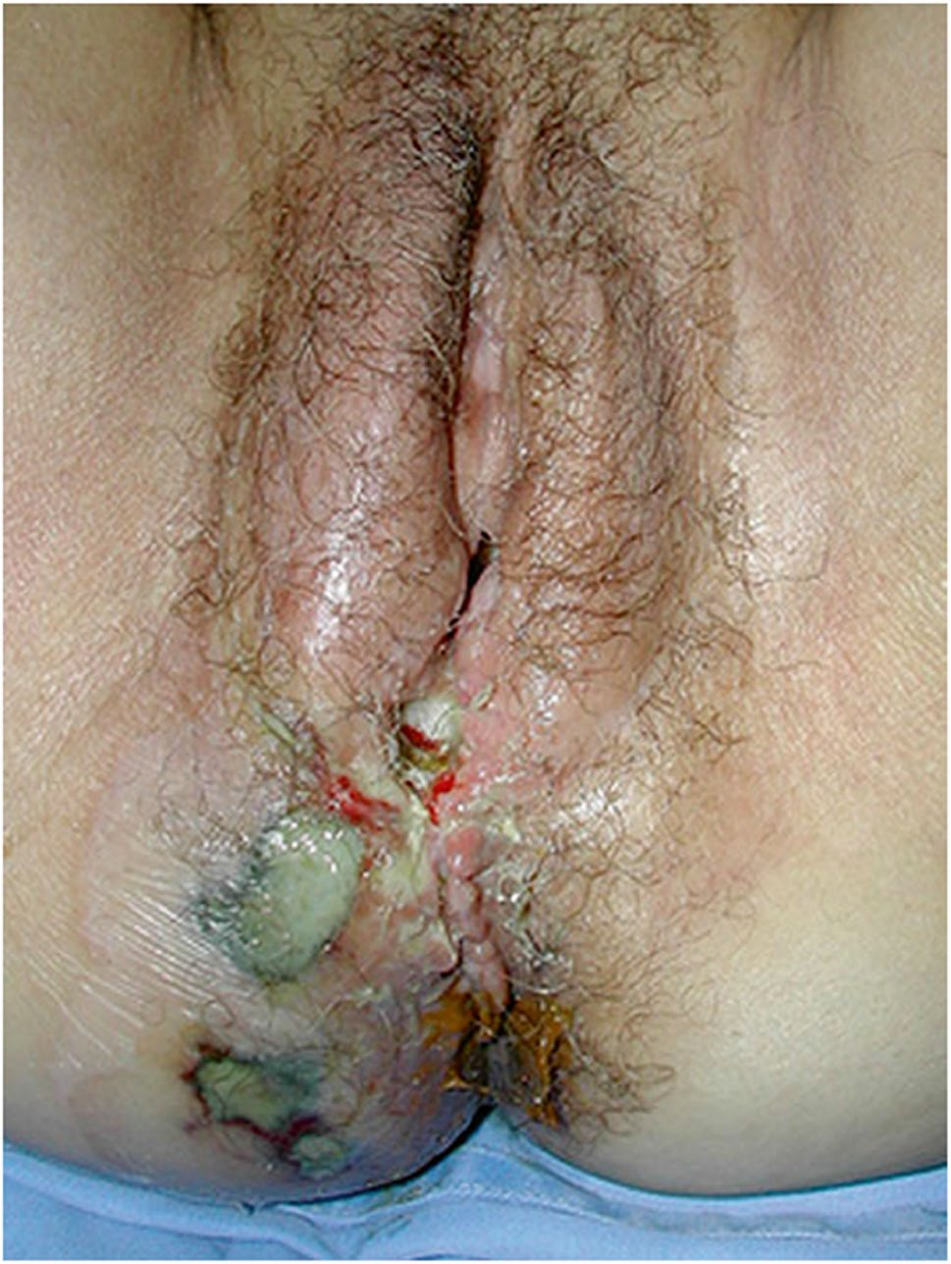
Necrotizing perineal infection [79]. *Source:* Reprinted by permission from Springer Nature

A surgical infection of the perineal wound, associated bleeding, pain, offensive discharge and signs of Redness, Edema, Ecchymosis, Discharge and disruption of wound edge Approximation (REEDA) [78] (Fig. 13). (NEW).

##### Perineal/vulval cellulitis:

Bacterial infection involving the inner layers of the perineal and vulval skin. (NEW).

##### Necrotizing fasciitis:

**Fig. 14:**
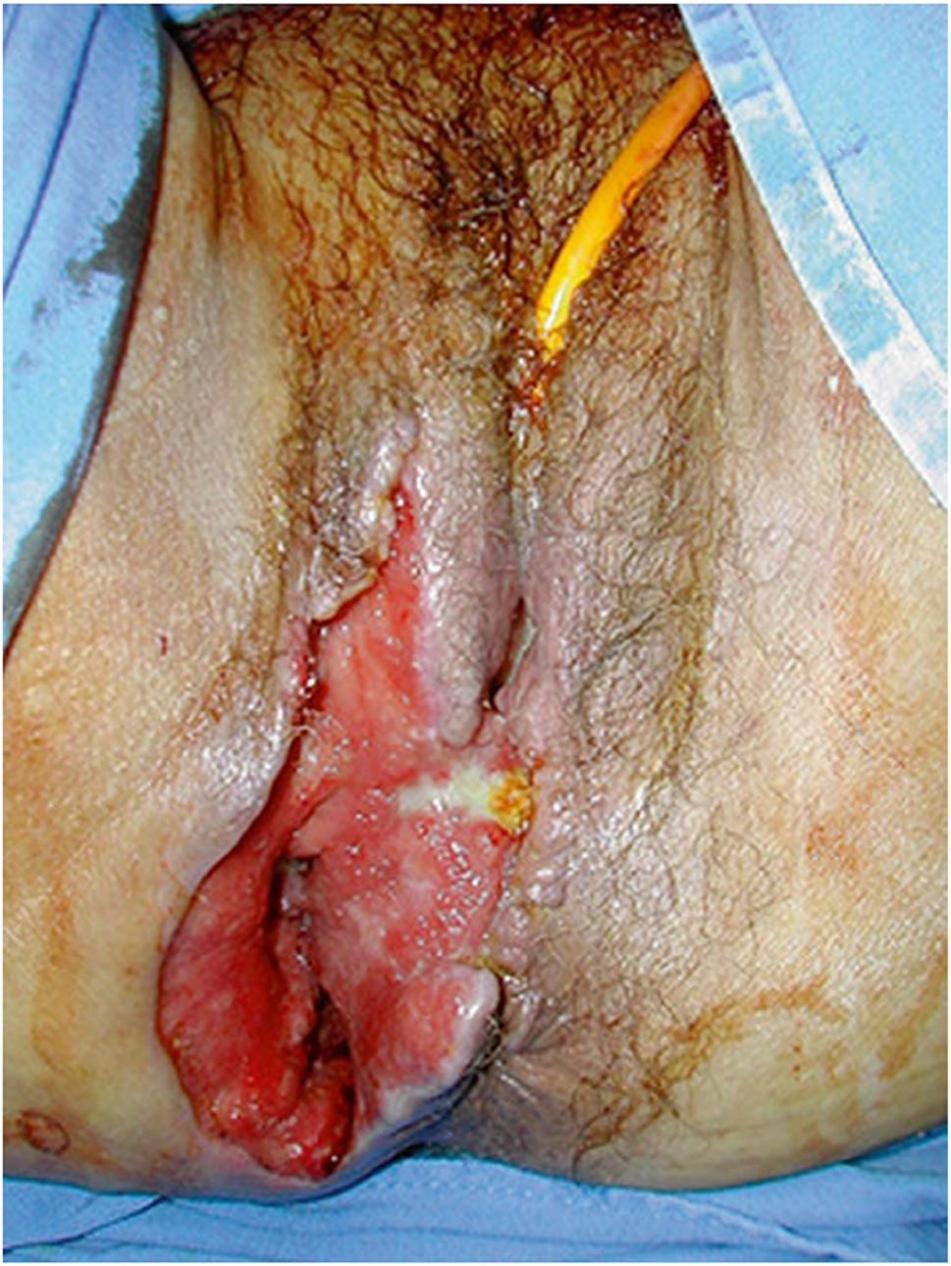
Debridement of necrotizing perineal infection [79]. *Source:* Reprinted by permission from Springer Nature

A severe soft tissue infection that is caused by bacteria, and is marked by oedema and necrosis of subcutaneous tissues with involvement of adjacent fascia and by painful red swollen skin over affected areas. It is associated with sepsis and the associated systemic inflammatory response syndrome causing changes in biochemical or hematologic parameters, but may be difficult to distinguish from cellulitis, abscess or other soft tissue infection, in the early stages. Risk scoring tools such as the Laboratory Risk Indicator for Necrotizing Fasciitis score indicating systemic toxicity, may assist in identifying those at intermediate (score 6–7) or high (≥ 8) risk which warrant urgent surgical evaluation and debridement [80] (Fig. 14). (NEW).

##### Postpartum perineal wound dehiscence (breakdown):

A breakdown of the suture line resulting in a dehiscence of a perineal wound during the postpartum period and up to 12 months after delivery. (NEW). _FN4.4_

#### Vulval Complications

##### Labial scarring and defects:

Labial tissues are scarred, distorted or asymmetrical following obstetric trauma and repair. (NEW).

##### Vulval fusion (agglutination):

Labial lips fused. Vulval/labial fusion is a spontaneous approximation of lacerations of the labia resulting in distorted anatomical healing, dyspareunia or obliteration of the introitus. (NEW).

##### Introital narrowing:

Vaginal entry or penetration is difficult or impossible (penis or sexual device). (NEW).

#### Levator ani muscle injuries

##### Levator avulsion:

The disconnection of the muscle from its insertion on the inferior pubic ramus and the pelvic sidewall, and may be complete, partial, unilateral or bilateral. Avulsion is a common consequence of overstretching of the levator ani during the second stage of labor and is detectable on palpation. Complete and partial defects may be diagnosed on tomographic TVUS or TPUS modalities using the criteria defined in the imaging section or on MRI. Defects are usually visualized most clearly on maximal PFMC [81]. Levator ani injuries affect the size of the levator hiatus and are associated with the development of anatomical and symptomatic prolapse [9].

Levator avulsions can be:

##### Complete levator avulsion:

Complete detachment of the levator ani muscle from its insertion to the inferior pubic ramus (Type II defect). (NEW).

##### Partial levator avulsion:

Partial detachment of the levator ani muscle from its insertion to the inferior pubic ramus (Type I defect) [82]. (see 3.3.3.5.3) (NEW).

#### Hiatal ballooning

Excessive distensibility of the levator hiatus. Levator ani injuries affect the size of the levator hiatus, with a hiatal enlargement to over 25 cm ^2^ on straining manoeuvre defined as “ballooning”, and are related to symptoms and signs of prolapse [8]. (NEW).

#### Pelvic organ prolapse

The diagnosis by symptoms and clinical examination (POP-Q), assisted or not by any relevant imaging, of descent of one or more of the anterior vaginal wall (central, paravaginal or combination cystocele), posterior vaginal wall (rectocele), the uterus (cervix) or the apex of the vagina (vaginal vault or cuff scar after hysterectomy), or the perineum (perineal descent). The presence of any such sign should correlate with relevant POP symptoms [6]. (CHANGED). _FN4.5_

#### Postpartum vaginismus

De novo recurrent or persistent spasm of vaginal musculature that interferes with vaginal penetration in the postpartum period and up to 12 months after delivery. (NEW).

### Urinary tract diagnoses [83]

#### Voiding dysfunction

A diagnosis by symptoms and investigations, including urodynamics, is defined as abnormally slow and/or incomplete micturition, based on abnormally slow urine flow rates and or abnormally high post-void residuals, ideally on repeated measurement to confirm abnormality. Pressure-flow studies can be required to determine the cause of the voiding dysfunction [8, 14]. _FN4.6_

##### Postpartum urinary retention (PPUR):

Inability to empty the bladder completely during the postpartum period and up to 12 months after delivery characterized by high PVR accompanied or not by symptoms of bladder distension. There is a generally (but not always) evidence of painless and palpable or percussible bladder in the postpartum period and up to 12 months after delivery, suggestive of a chronic high PVR. The patient experiences slow flow and incomplete bladder emptying. (NEW).

##### Acute Postpartum urinary retention (APPUR):

A patient, with or without symptoms of bladder distension, is unable to pass any urine despite having full bladder, which on examination is painfully distended and readily palpable or percussible and on catheterization is characterized by high PVR during the postpartum period and up to 12 months after delivery [7]. (NEW).

##### Postpartum voiding dysfunction—Retention with overflow:

Involuntary loss of urine directly related to an excessively full bladder in retention [7]. (NEW).

##### Postpartum chronic urinary retention:

Complaint of chronic or repeated inability to empty the bladder, despite the ability to pass some urine. This may result in the frequent passage of small amounts of urine or urinary incontinence and a distended bladder [7]. (NEW). _FN4.7_

##### Postpartum acute on chronic retention:

An individual with chronic retention goes into acute retention and is unable to void [7]. (NEW).

##### Covert postpartum urinary retention:

Diagnosis of high PVR with no or minimal voiding symptoms and a postvoid residual volume greater than 100 ml, in the early postpartum period, up to 4 weeks [28, 68, 69]. (NEW). _FN4.8_

#### Postpartum sexual dysfunction

A de novo postdelivery (in the postpartum period and up to 12 months after delivery) diagnosis of an abnormality or difficulty with sexual intercourse, experienced by the woman or partner, confirmed by clinical history and/or signs. The diagnosis may be associated with vaginal, urinary, anorectal, prolapse, pain symptoms, and may include decreased libido, arousal or anorgasmia. This diagnosis may persist or develop to meet DSM V criteria of Female Sexual Interest/Arousal Disorder (FSIAD) and/or DSM IV criteria of genito-pelvic pain/penetration disorder (GPPPD), and/or Female Orgasmic Disorder [8, 12, 84]. (NEW).

#### Postpartum Sexual Interest/Arousal disorder

Lack of, or significantly reduced, sexual interest/arousal during the postpartum period and up to 12 months after delivery as manifested by 3 of the following:

1. Absent/reduced interest in sexual activity
2. Absent/reduced sexual/erotic thoughts or fantasies
3. No/reduced initiation of sexual activity and unreceptive to partner’s attempts to initiate
4. Absent/reduced sexual excitement/pleasure during sexual activity in almost all or all (75%–100%) sexual encounters.
5. Absent/reduced sexual interest/arousal in response to any internal or external sexual/erotic cues (written, verbal, visual).
6. Absent/reduced genital or non-genital sensations during sexual activity in almost all or all (75%–100%) sexual encounters. (NEW).

#### Postpartum Genito-Pelvic Pain/Penetration disorder

Persistent or recurrent difficulties during the postpartum period and up to 12 months after delivery with 1 or more of the following:

1. Vaginal penetration during intercourse
2. Marked vulvovaginal or pelvic pain during intercourse or penetration attempts
3. Marked fear or anxiety about vulvovaginal or pelvic pain in anticipation of, during, or as a result of vaginal penetration
4. Marked tensing or tightening of the pelvic floor muscles during attempted vaginal penetration. (NEW).

#### Postpartum orgasmic disorder

Presence of either of the following on all or almost all (75%–100%) occasions of sexual activity during the postpartum period and up to 12 months after delivery:

1. Marked delay in, marked infrequency of, or absence of orgasm.
2. Markedly reduced intensity of orgasmic sensations. (NEW).

### Postpartum vaginal hematomas

These diagnoses can be confirmed using imaging modalities.

#### Postpartum infralevator hematomas 

**Fig. 15:**
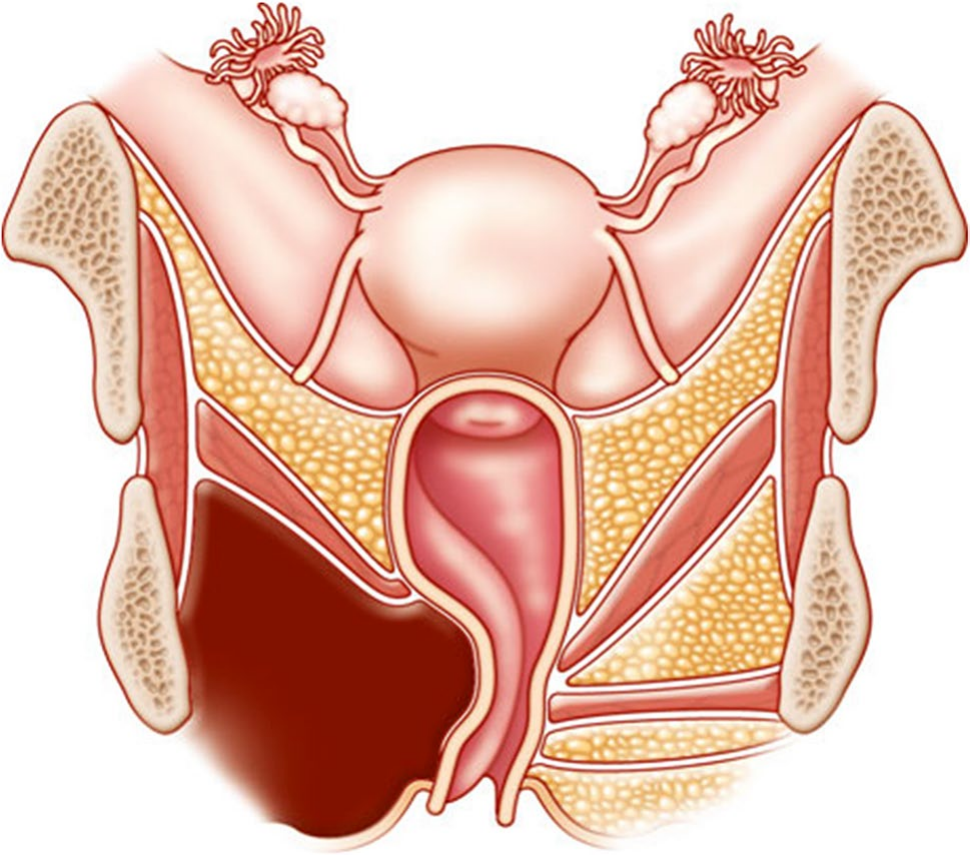
Infralevator hematoma [79]. *Source:* Reprinted by permission from Springer Nature

Are defined as hematomas (blood collections) in the infralevator space occurring following childbirth and associated with massive swelling and ecchymosis of the labia, perineum, and lower vagina with severe vulval and perineal pain. Anorectal tenesmus may result from extension into the ischiorectal fossa, while urinary retention may result from spread ventrally into the paravesical space (Fig. 15). (NEW).

#### Postpartum supralevator hematoma 

**Fig. 16:**
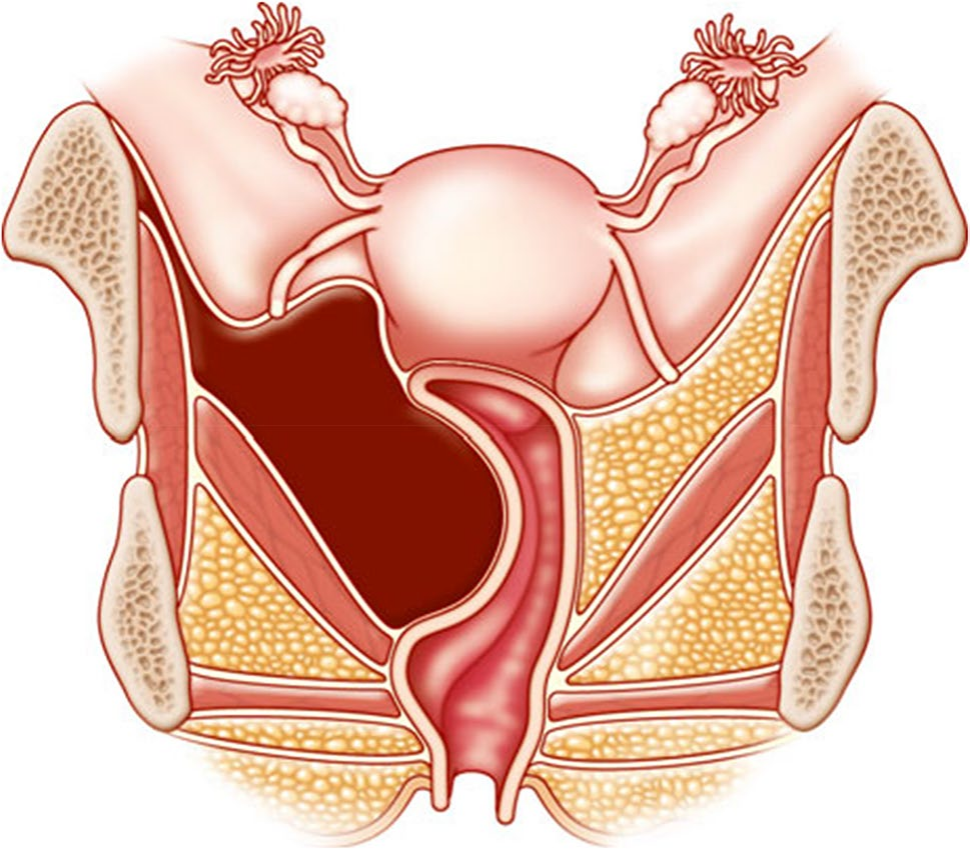
Supralevator hematoma [79]. *Source:* Reprinted by permission from Springer Nature

Is a hematoma in the supralevator space occurring following childbirth. It can be palpable as a rubbery mass protruding into the vaginal wall and potentially occluding the vaginal and causing pain and pressure symptoms (Fig. 16). (NEW).

#### Vulval hematoma

 Is a blood collection subcutaneously in the vulval area and usually results from injuries to the pudendal artery or its branches during childbirth. (NEW).

### Postpartum pain

#### Postpartum vaginal pain syndrome

The occurrence of persistent or recurrent episodic vaginal pain following childbirth, during the postpartum period and up to 12 months after delivery. There is no proven vaginal infection or other obvious pathology [15, 85]. (NEW).

##### Chronic (or persistent) vaginal pain:

Chronic pelvic pain during the postpartum period and up to 12 months after delivery, characterized by persistent pain lasting longer than 6 months or recurrent episodes of abdominal/pelvic pain, hypersensitivity or discomfort often associated with elimination changes, and sexual dysfunction often in the absence of organic etiology. (NEW).

##### Postpartum somatic nerve pain:

Nerve injury (stretching, blunt trauma, compression, entrapment, suture ligature) during the postpartum period and up to 12 months after delivery. (NEW).

##### Postpartum pudendal neuralgia:

Pudendal neuralgia is a disabling form of pelvic pain during the postpartum period and up to 12 months after delivery. It is related to a ligamentous nerve compression mechanism. This pain is associated with the second stage of labor, vaginal injuries and repairs.

1. Unilateral or bilateral.
2. Lancinating burning pain in the clitoris, urethra, labia, perineum and/or anus.
3. Worse with sitting.
4. Relieved by standing or supine position. (NEW).

#### Postpartum chronic pelvic joint, ligament or bone pain syndrome

Complaint of: 1. Joint pain: i. Sacroiliac or pubic symphysis joint. 2. Ligament pain: i. Sacrospinous or sacrotuberous ligament. 3. Bony pain: i. Pain described in or along the margins of the pubic ramus, ilium, ischial spine or ischial tuberosity during the postpartum period and up to 12 months after delivery. (NEW).

### Postpartum anorectal complications

#### Postpartum anal incontinence

Involuntary loss of flatus and/or solid or liquid stool during the postpartum period and up to 12 months after delivery. (NEW). _FN4.9,FN4.10_

### Obstetric pelvic floor nerve trauma

#### Obstetric neuropathy

Disease or dysfunction of one or more peripheral nerves, secondary to childbirth. (NEW).

#### Obstetric pudendal nerve injury

Injury to the pudendal nerve or its branches during vaginal childbirth. (NEW).

**Footnotes for Section** **Diagnoses**

**4.1 RCOG classification for perineal and anal sphincter injuries:**

Define perineal or genital trauma caused by either tearing or episiotomy as follows:

- first degree—cutaneous trauma to the vaginal epithelium or perineal skin only
- second degree—injury to the perineal muscles but not the anal sphincter
- third degree—injuryto the perineum involving the anal sphincter complex:
  - 3a—less than 50% of external anal sphincter thickness torn
  - 3b—50% or more of the external anal sphincter thickness torn
  - 3c—internal anal sphincter torn.
- fourth degree—injury to the perineum involving the anal sphincter complex (external and internal anal sphincter) and anal epithelium.

**4.2:** For a more detailed definition see ICS report on the terminology for female pelvic organ fistulas [10]. The past two commonly used classification systems have been the Waaldijk and Goh classifications [86].

**4.3:** Hemorrhoids: Abnormality of the normal cushion of specialized, highly vascular tissue in the anal canal in the submucosal space. Hemorrhoids can be divided into those originating above the dentate line which are termed internal and those originating below the dentate line which are termed external. Internal hemorrhoids are graded as follows:

- Grade I—bleeding without prolapse.
- Grade II—prolapse with spontaneous reduction.
- Grade III—prolapse with manual reduction.
- Grade IV—incarcerated, irreducible prolapse.

Grade II and Grade III hemorrhoids will become evident on asking the patient to bear down and grade IV hemorrhoids are obvious at the time of the examination. A proctoscopy is essential in examining for hemorrhoids unless they are completely prolapsed.

**4.4: Wound dehiscence:** A bursting open, splitting or gaping along natural or sutured lines [87].

**4.5:** For a more detailed definition see IUGA/ICS joint report on the terminology for Female Pelvic Organ Prolapse [8].

**4.6:** for a more detailed definition see IUGA/ICS joint report on the Terminology for Female Pelvic Floor Dysfunction [6].

**4.7:** Persistent postpartum urinary retention has been defined as the inability to void spontaneously by the third day postpartum despite the use of intermittent catheterization [88].

Non-neurogenic chronic urinary retention (CUR) has been proposed to refer to elevated post-void residual volumes of greater than 300 mL that has persisted for at least 6 months and is documented on two or more separate occasions [89].

**4.8:** PVRs over 100 mls in women with symptoms of lower urinary tract dysfunction are regarded as high. PVRs above 100 mls in the early postpartum period (up to 4 weeks) should be monitored; after 4 weeks further assessment should be considered depending on symptoms.

**4.9:** Postpartum anal incontinence could be due to:

1. Anal sphincter disruption of the external anal sphincter, internal anal sphincter or both;
2. Hypocontractile/acontractile sphincter due to pudendal nerve neuropathy;
3. Combined anal sphincter disruption and hypocontractile/acontractile sphincter;
4. Rectal overactivity due to exaggerated smooth muscle contraction of the rectum could also be associated with hypersensitivity;
5. Overflow incontinence seepage of stool due to fecal impaction [9].

**4.10:** Anal incontinence is usually most prevalent after one month postpartum and resolving almost completely by 1 year [90]. Overall prevalence decreases further in the next 6 years so that only a small percentage of women, especially with a history of operative delivery and/or anal sphincter injury, will complain of persistent anal incontinence 6 years later [91].

## Prediction of obstetric pelvic floor disorders and prevention

### Prediction of obstetric pelvic floor disorders

A process of prospectively evaluating the risk of sustaining pelvic floor trauma at childbirth. (NEW). _FN5.1_

#### Antenatal predictors

Pre-existing risk factors for the development of significant obstetric pelvic floor trauma, such as maternal age, BMI and bladder neck descent [92, 93]. (NEW).

#### Intrapartum predictors

Intrapartum risk factors for the development of significant obstetric pelvic floor trauma, such as the use of forceps and a prolonged second stage [94–97]. (NEW).

### Obstetric variable

A characteristic, quantity or attribute relating to childbirth that can be measured in research [98]. (NEW).

#### Obstetric risk factors

Obstetric variables associated with an increased risk of obstetric pelvic floor disorders [99] (NEW). _FN5.2_

### Primary prevention of obstetric pelvic floor trauma

Measures to prevent the occurrence of obstetric perineal trauma or postpartum pelvic floor dysfunction by avoiding or modifying risk factors (NEW).

#### Primary prevention of obstetric pelvic floor trauma before pregnancy

Lifestyle modifications; controlling diabetes mellitus, controlling body mass index (NEW).

#### Primary prevention of obstetric pelvic floor trauma during pregnancy

Lifestyle modifications, screening for gestational diabetes, ultrasound screening for fetal macrosomia, controlling weight gain, perineal massage, pelvic floor muscle training (controversial), induction labor for suspected macrosomia (controversial), elective cesarean section (NEW).

#### Primary prevention of obstetric pelvic floor trauma during labor/delivery

Maternal position, manual rotation of posterior position, avoidance of instrumental deliveries, preference for ventouse rather than forceps, performing a 60° mediolateral episiotomy (controversial), slowing the descent of the foetal head, manual perineal protection (controversial), warm compresses, perineal massage, bladder emptying before pushing, pushing without Valsalva (controversial), manual perineal support; or bundle of two or more of these measures (NEW).

#### Primary prevention of obstetric pelvic floor trauma after delivery

Avoiding urinary retention, lifestyle modifications, pelvic floor muscle training. (NEW).

#### Elective cesarean delivery

Delivery of the foetus via a cesarean (lower abdominal) incision prior to the onset of labor. It has been advocated as the only true primary prevention of perineal trauma strategy. Cesarean delivery after the onset of labor is not protective of trauma to the pelvic floor. (NEW).

#### Antenatal pelvic floor muscle training

Pelvic floor muscle exercises during the antenatal period. (NEW).

#### Perineal massage, antenatally or during second stage

A digital technique involving the insertion of one or two fingers into the vagina to a depth of 3–4 cm to massage the posterior vaginal wall in a U-shaped movement [100] to stretch the perineum and surrounding structures antenatally or during the second stage. (NEW).

#### Warm compresses in second stage

Application of a compress (pack) soaked in warm (38–44 degrees centigrade) water at the commencement of perineal stretching [101]. (NEW).

#### Maternal position during delivery

The position adopted by women in the second stage of labor and may be associated with the risk of perineal trauma. These may be defined as:

- Upright: sitting, standing, semi-recumbent at > 45 degrees to the horizontal, kneeling, squatting, all fours, walking.
- Recumbent: supine, semi-recumbent at < 45 degrees to the horizontal, lateral, Trendelenburg, lithotomy, knee-elbow [102]. (NEW).

#### Water immersion during labor and birth

Immersion in water by a pregnant woman during any stage of labor (first, second, third) where the woman’s abdomen is completely submerged. ‘Waterbirth’ refers to where the fetus is born under the water [103]. (NEW).

#### Avoidance of use of forceps

A primary or secondary preventive measure of reducing the risk of levator injury, [104] obstetric anal sphincter injury [4] and pelvic floor dysfunction when compared with other modes of delivery. (NEW).

#### Manual Perineal support (protection) at birth

**Fig. 17:**
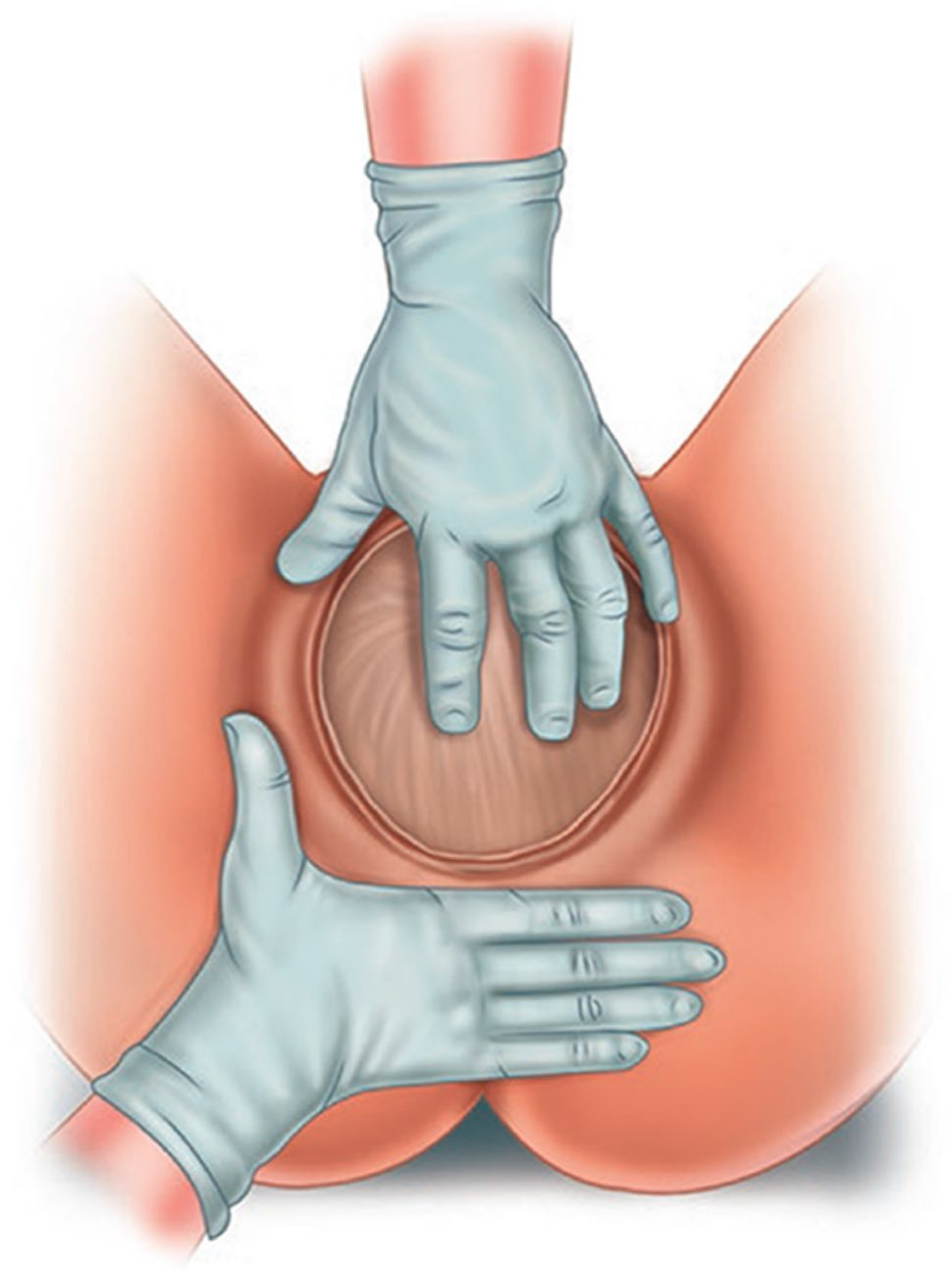
Central palmar support Reprinted/adapted by permission from Springer Nature Customer Service Centre GmbH Springer Nature: Perineal Mapping. in Perineal Trauma at Childbirth (KMK Ismail) [COPYRIGHT] (2017) [105]

**Fig. 18:**
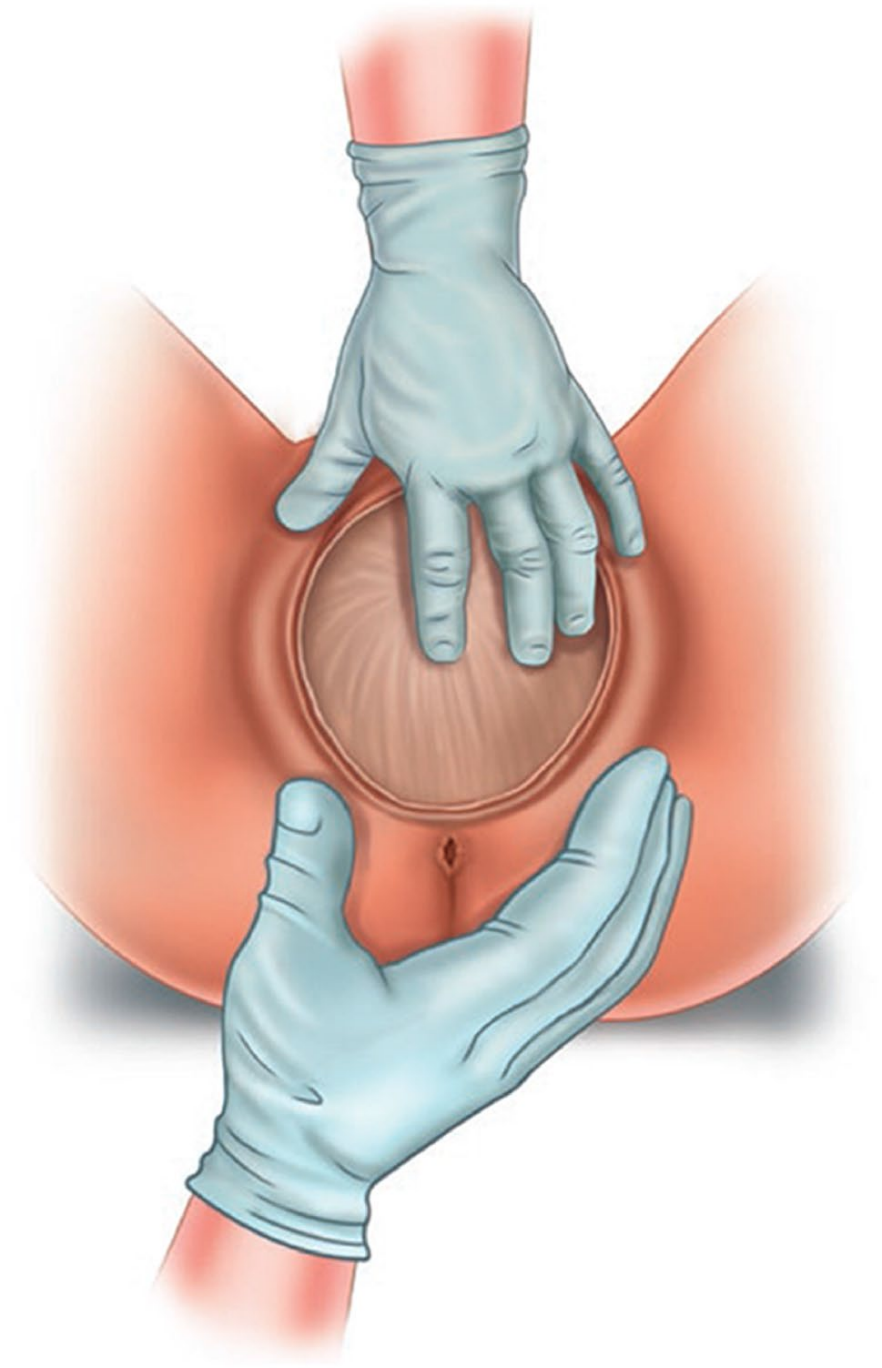
Viennese method. Reprinted/adapted by permission from Springer Nature Customer Service Centre GmbH Springer Nature: Perineal Mapping. in Perineal Trauma at Childbirth (KMK Ismail) [COPYRIGHT] (2017) [105]

**Fig. 19:**
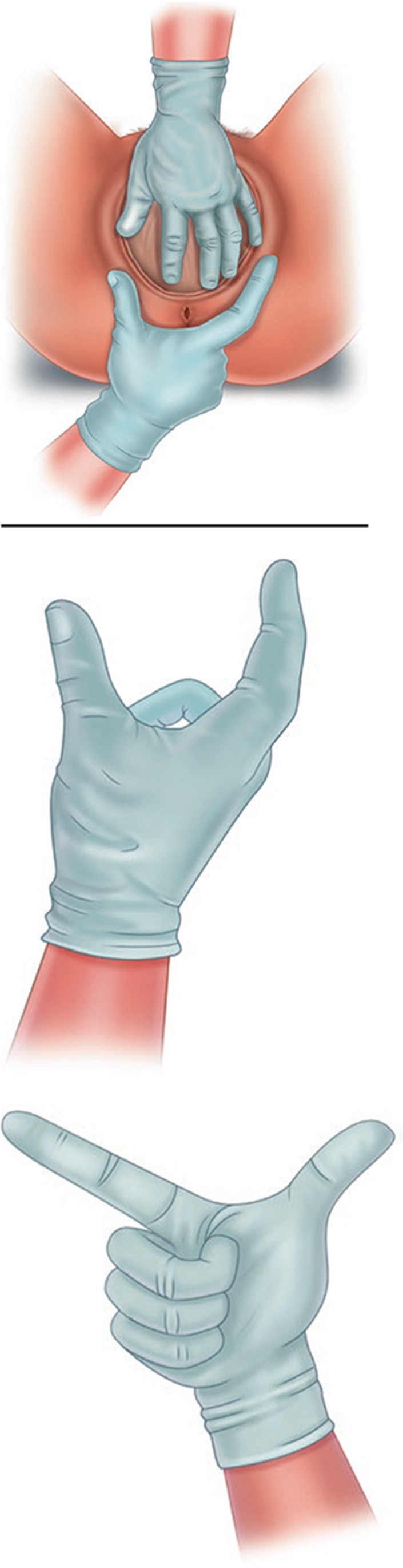
Finnish method. (**a**) Finnish method—application of the accoucheur’s hands on the fetal head and perineum; (**b**) Finnish method—accoucheur’s dominant hand, view from above; (**c**) Finnish method—accoucheur’s dominant hand, view from the front Reprinted/adapted by permission from Springer Nature Customer Service Centre GmbH Springer Nature: Perineal Mapping. in Perineal Trauma at Childbirth (KMK Ismail) [COPYRIGHT] (2017) [105]

A bimanual technique that requires support of the posterior fourchette with one hand and cupping of the foetal head with the other to prevent the head coming out with great force as it progresses at crowning. (Figs. 17, 18 and 19) (NEW).

#### Bearing down in labor

A common technique during second stage involving closed-glottis pushing (holding breath while pushing) duration of 10 s or more. This is contrasted with other spontaneous breathing techniques while pushing [106]. (NEW).

#### Delayed pushing in second stage

Once fully dilated, a delay in the onset of pushing would allow spontaneous descent and rotation of the foetal head to increase efficiency of pushing efforts and reducing the risk of the parturient fatigue and instrumental delivery [106]. (NEW).

#### Perineal hyaluronidase injection in second stage

The injection of hyaluronidase to the perineum to relax the connective tissue around the skin or subcutaneous muscles and render them less vulnerable to mechanical stress or extension during the passage of the fetus through the vaginal canal [107]. (NEW).

### Secondary prevention of obstetric pelvic floor trauma

Measures to reduce severity of obstetric perineal trauma or postpartum pelvic floor dysfunction on patients with known risk factors or with mild to moderate symptoms. (NEW).

#### Secondary prevention of obstetric pelvic floor trauma before pregnancy

Lifestyle modifications controlling diabetes mellitus, controlling body mass index (NEW). _FN5.3_

#### Secondary prevention of obstetric pelvic floor trauma during pregnancy

Lifestyle modifications, screening for gestational diabetes, ultrasound screening for fetal macrosomia, controlling weight gain, perineal massage, pelvic floor muscle training (controversial), induction of labor for suspected macrosomia (controversial) [108]. (NEW).

#### Secondary prevention of obstetric pelvic floor trauma during labor/delivery

Maternal position, manual rotation of turn posterior position, avoidance of instrumental deliveries, preference for ventouse rather than forceps, performing a 60° mediolateral episiotomy (controversial), slowing the descent of the foetal head, manual perineal protection (controversial), warm compresses, perineal massage, bladder emptying before pushing, pushing without Valsalva (controversial), manual perineal support, or bundle of two or more of these measures (NEW).

#### Secondary prevention of obstetric pelvic floor trauma after delivery

Avoiding urinary retention, lifestyle modifications. (NEW).

#### Episiotomy

Episiotomy is a surgical enlargement of the vaginal orifice by an incision to the perineum during the last part of the second stage of labor during vaginal delivery [109]. (NEW).

##### **Types of** episiotomy:

**Fig. 20:**
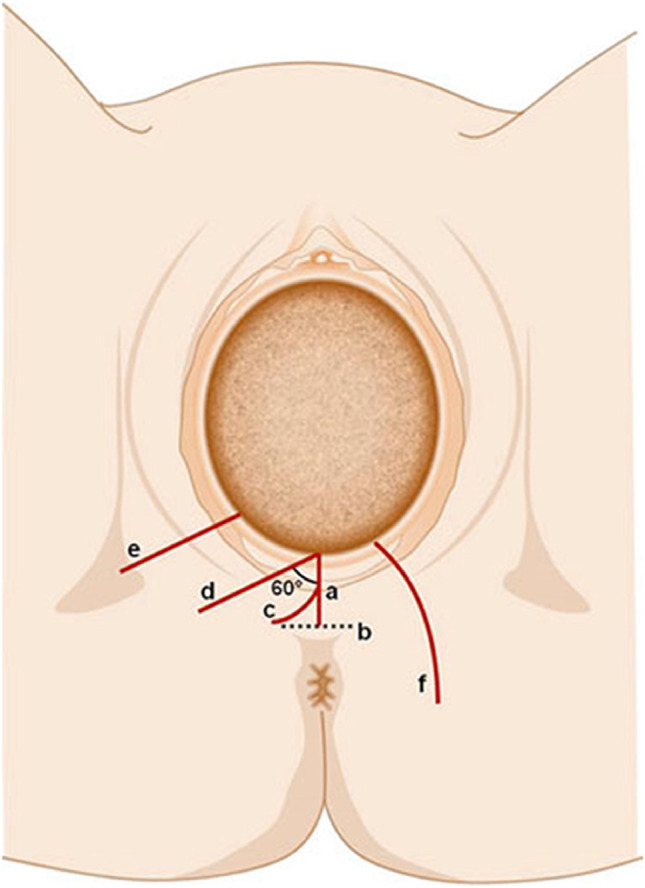
Types of episiotomy [110]. Key: *a* midline episiotomy; *b* modified median episiotomy; *c* J-shaped episiotomy; *d* mediolateral episiotomy; *e* lateral episiotomy; *f* radical lateral (Schuchardt incision). *Source:* Reprinted by permission from Springer Nature

Main types of episiotomy include median, modified median, J shaped, mediolateral, lateral, radical lateral, and anterior (Fig. 20).

##### Median (midline, medial) episiotomy:

Median episiotomy starts at the posterior fourchette and runs along the midline through the central tendon of the perineal body *. The origin of the initial incision is within 3 mm of the midline in the posterior fourchette and the direction of the cut is between 0° and 25° of the sagittal plane [111]. The extension of the incision is half of the length of the perineum [112]. (NEW).

##### Modified median episiotomy:

This is a modification of median episiotomy. It involves extending the median episiotomy by adding two transverse incisions bilaterally just above the anal sphincter [113]. The transverse incisions are perpendicular to the midline, 2–5 cm in total length. This type of episiotomy aims to increase the diameter of the vaginal outlet by 83% compared with a standard median episiotomy, possibly by separation of the perineal membrane and sphincter attachments [114]. The origin of the initial incision is within 3 mm of the midline in the posterior fourchette and the direction of the cut is between 0° and 25° of the sagittal plane, with two transverse cuts on each side added [111]. (NEW).

##### ‘J’-shaped episiotomy:

This type starts with a midline incision and is then curved laterally to avoid the anus. In this technique curved scissors are used starting in the midline of the vagina until the incision is 2–5 cm from the anus. Then the ‘J’ is made by directing the incision towards the ischial tuberosity away from the anal sphincter. The origin of the initial incision is within 3 mm of the midline in the posterior fourchette and the direction of the cut is at first midline, then ‘J’ is directed towards the ischial tuberosity [111]. (NEW).

##### Mediolateral episiotomy:

This type of episiotomy involves an incision beginning in the midline and directed laterally and downwards avoiding the anal sphincter [115]. The origin of the initial incision is within 3 mm of the midline in the posterior fourchette and the direction of the cut is laterally at an angle of at least 60° from the midline towards the ischial tuberosity [111]. (NEW).

##### Lateral episiotomy:

This type of episiotomy begins laterally to the vaginal introitus and is directed towards the ischial tuberosity [112]. The origin of the initial incision is more than 10 mm from the midline in the posterior fourchette and the direction of the cut is laterally towards the ischial tuberosity [111]. (NEW).

##### Radical lateral (Schuchardt incision):

Radical lateral episiotomy is often considered to be a non-obstetrical incision. It is a fully extended episiotomy, deep into one vaginal sulcus and is curved downwards and laterally part way around the rectum. It may be performed at the beginning of radical vaginal hysterectomy or trachelectomy to permit easy access to the parametrium, or rarely to facilitate complicated deliveries (large head, difficult breech or for management of shoulder dystocia). The origin of the initial incision is more than 10 mm from the midline and the direction of the cut is laterally towards the ischial tuberosity and around the rectum [111]. (NEW).

##### Anterior episiotomy (deinfibulation):

The anterior episiotomy or deinfibulation is defined as a surgical incision usually performed during delivery in women who previously had infibulation [116]. Fused labia minora are incised in the midline anteriorly until the level of the external urethral meatus. The clitoris and surrounding tissues or clitoral remnants should not be incised. The origin of the initial incision is midline and the direction of the cut is midline, directed towards the pubis [111]. (NEW).

### Tertiary prevention of obstetric pelvic floor trauma

Measures to manage women with previous obstetric trauma or severe pelvic floor dysfunction and attempt to treat or prevent further complications.

- Lifestyle modifications
- Mode of delivery in subsequent pregnancies for women with previous obstetric trauma or severe pelvic floor dysfunction (i.e. fecal incontinence). (NEW).

**Footnotes for Section** **Prediction of obstetric pelvic floor disorders and prevention**

**5.1 Risk prediction model**: a mathematical equation that uses a number of predictors to estimate the probability of obstetric pelvic floor trauma. It can assist in clinical decision making for clinicians and patients. Before being used in clinical practice, risk prediction models should be validated.

Recent risk prediction models for obstetric pelvic floor trauma include OSIRIS and UR-CHOICE [117–120]. (NEW).

**5.2** Instrumental delivery, especially forceps, midline episiotomy, and a persistent occiput posterior position, have been associated with the highest risk of developing severe perineal trauma [99].

**5.3** Lifestyle modifications: Interventions that intentionally change the way a person lives in order to improve health status to prevent or avoid deterioration of any pelvic floor disorders (weight loss, avoid heavy lifting or coughing, cease tobacco smoking, avoid or treat constipation, modify occupational health parameters, avoid physical straining or heavy weight lifting at work).

## Management of obstetric pelvic floor trauma

### Conservative treatments

Restricted to non-surgical and non-pharmacological treatment. (NEW).

#### Conservative treatment of vaginal/perineal tears

The non-surgical treatments that allow spontaneous healing of surgical trauma, including topical hygiene measures (avoiding irritant soaps, detergents, and douches) and expectant management. (NEW). _FN6.1_

##### Vulval hygiene:

Involves maintaining a clean perineum by means of washing the area on a regular basis and wearing cotton underwear. To avoid vulval irritation, shampoo, perfumed creams, or soap should be avoided [55].

##### Anal hygiene:

Involves keeping the perianal region clean, which is especially important when fecal seepage is present. Advice includes using soft toilet paper or moist wipes (avoiding any with an alcohol base), always wiping from front to back, washing after a bowel movement, then gently patting dry [55].

##### Perineal cryotherapy:

The application of substances that remove body heat and reduce the temperature of the tissues as a treatment approach [121]. (NEW).

##### Sitz baths:

Warm bath to which salt has been added [122]. (NEW).

##### Pelvic brace/belt:

 Tubigrip or trochanteric belts worn over the lower abdomen and pelvic area, just cranial to the greater trochanters. Exerts a small amount of force and aids in restoration and stability of the pelvic ring [123]. (NEW).

#### Pudendal Nerve Infiltration

Infiltration of local anesthetic like bupivacaine and corticosteroid around the pudendal nerve to provide symptom relief in cases of pudendal neuralgia [124].

#### Physical Therapies

##### Pelvic physiotherapy:

Assessment, prevention and/or treatment of pelvic floor dysfunction, performed by a pelvic physiotherapist. The therapy aims at reducing pelvic floor symptoms and related bother as well as improvement of pelvic floor function. Pelvic physiotherapy covers many specialized therapies that can be used to train the pelvic floor: physical activity, cognitive behavioral therapy, bladder training, bowel habit training, muscle training (endurance, power), coordination training, biofeedback, and electrical muscle stimulation. The physiotherapist can use adjuvant treatments such as soft tissue therapies. _FN6.2_ For the treatment of obstetric trauma associated POP it may be useful to associate the use of devices _FN6.3_ with physiotherapy.

### Surgical Management

#### Surgical repair of vaginal and perineal trauma

Surgical treatment of a tear by suturing and closure of the anatomical defect. (NEW). _FN6.4_ (See Appendix).

#### Levator ani repair

Dissection from the ischial spine to the pubic bone and suturing of the various divisions of the levator ani muscle to recreate a functioning levator plate [125]. (NEW).

#### Perineoplasty

Surgical procedure intended to narrow genital hiatus, reduce introital gap and increase perineal body. It can be performed as a stand-alone procedure or in combination with other perineal or vaginal repairs [126]. (NEW).

#### Perineal scar revision

Surgical excision of symptomatic perineal scar. It will usually include resuturing to create a new scar. (NEW).

#### Fenton’s procedure

Surgical procedure to increase genital hiatus and widen the introitus by excising scar tissue and/or an area of constriction at the entrance of the vagina. (NEW).

#### Z-plasty procedure to treat introital stenosis

Involves a central incision along the length of the constriction and 2 lateral incisions at an angle of 60° to form a Z as shown in the video. The lengths of the three limbs and the angles formed between the central and lateral limbs are equal. This creates two triangular tissue flaps which when transposed change the length as well as orientation of the scar. This is associated with a 40% gain in functional length along the central incision once the flaps are transposed. (NEW). _FN6.5_

**Footnotes for Section** **Management of obstetric pelvic floor trauma**

**6.1** Whilst most practitioners tend to suture vaginal and perineal tears, the debate of whether to leave the skin unsutured has been longstanding. Overall, there does not seem to be enough consistent evidence to support a change in practice of leaving perineal cutaneous trauma unsutured.

**6.2 Soft Tissue Therapies consist of:**

**Touch desensitization:** The manipulation of the soft tissues of the body for the purpose of affecting the nervous, muscular, respiratory, and circulatory systems [55].

**Massage**: The manipulation of the soft tissues of the body for the purpose of affecting the nervous, muscular, respiratory, and circulatory systems [55].

**Abdominal massage**: Therapist or self-directed massage of the abdominal wall with the aim of stimulating peristalsis and relieving the symptoms of constipation. Generally, the technique follows the ascending, transverse, and descending colon to aid emptying. The effect may be mechanical or sensory [55].

**Myofascial release techniques**: The use of deep friction and stroking of the fascia of the body to improve the ability of the fascia to deform and move within the body [55].

**Skin rolling**: A manual technique in which skin is pulled away from the underlying structures and elongated in various directions [55].

**Scar massage**: A specific application of soft-tissue mobilization to an adherent scar [55].

**Perineal massage**: intravaginal massage by the woman, her partner, or the clinician. Technique includes alternating downward and sideward pressure, using thumb and forefinger and a natural oil, with the aim of stretching and elongating the tissue in preparation for vaginal childbirth, or for treatment of adherent scarring in the perineum [55].

**Transverse friction**: the operator’s fingertip is placed on the exact site of the lesion and rubbed firmly across the direction of the fibers of the affected tissue [55].

**Thiele’s massage**: per-rectal digital massage of the levator ani, sweeping lengthwise along the muscle fibers. Massage is begun lightly, and pressure is increased as tenderness decreases [55].

**TrP treatment:** (sometimes called myofascial trigger point treatment): Soft-tissue mobilization specifically targeting trigger points and may include ischemic pressure, massage, myofascial release, electrotherapy, ultrasound, laser, spray and-stretch, injection (a variety of chemicals including local anesthetic, botulinum toxin or steroids), dry needling (insertion of a solid needle into the TrP), and stretching [55].

**6.3 Devices for the treatment of obstetric trauma associated POP fundamentally are vaginal pessaries** defined as a device that is inserted into the vagina to provide structural support to one or more of descending vaginal compartments, i.e., the uterus, anterior vaginal wall (and bladder), posterior vaginal wall (and rectum) [127].

Vaginal pessaries can be broadly divided into two types:

- **support pessaries** Ring pessary with or without central support; Gehrung, Hodge pessaries.
- **Space filling pessaries** Donut; cuboid; Gellhorn; inflatable; shelf (similar to a Gellhorn but asymmetric) [8].

The most frequently used pessaries are [127]:

- Ring pessary with or without central support
- Gellhorn pessary; round solid pessary with a central stem
- Donut pessary
- Cuboid pessary
- Shelf pessary: Similar to a Gellhorn but asymmetric [8].

**6.4 Surgical repair of the anal sphincter**: Re-apposition of the injured external and/or internal anal sphincter. An incomplete external sphincter or internal sphincter injury may be repaired with an end-to-end technique, while a complete external sphincter injury may use an end-to-end or overlapping technique [128].

**Fig. 21:**
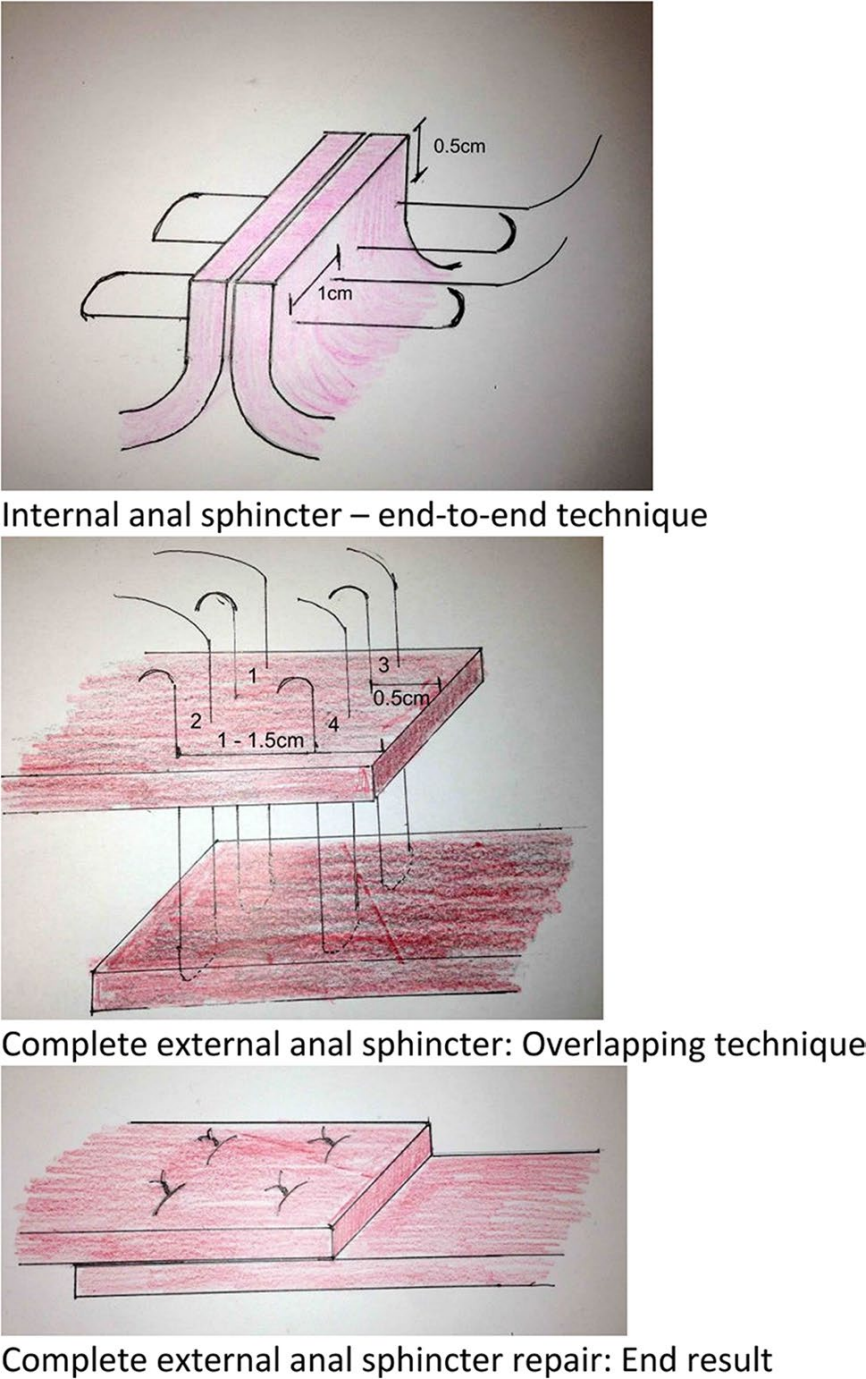
Repair of obstetric anal sphincter injuries [76]. *Source:* Reproduced with kind permission

**Fig. 22:**
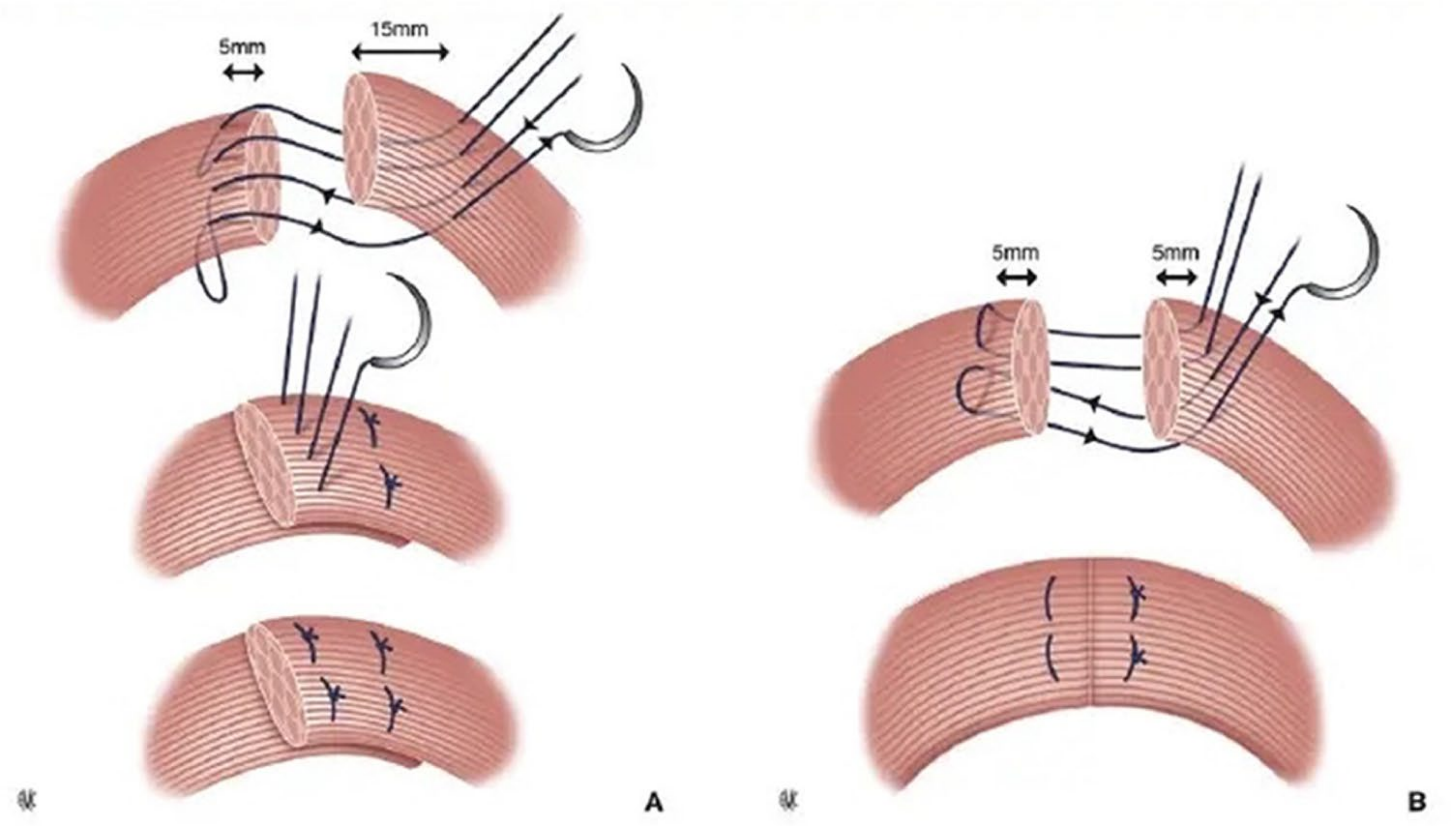
The two different methods of external anal sphincter repair. **A** Overlapping technique. **B** End-to-end technique. Extract from: V. Letouzey, E. Mousty, B. Fatton, J.-F. Bourgaux, M. Bertrand, M. Prudhomme, P. Marès, R. de Tayrac. Traumatisme anal chez la parturiente. EMC Gynecologie; Vol 11, n o 3, July 2016: pp 1–12. Copyright © 2016 Elsevier Masson SAS. All rights reserved. http://dx.doi.org/10.1016/S0246-1064(15)65086-1 [129]

An end-to-end technique involves apposition of the injured ends of the sphincter with interrupted horizontal mattress sutures. The overlapping technique involves using vertical mattress sutures to overlap one edge of the injured sphincter over the other (Figs. 21 and 22).

**6.5 Video:**

https://academy.iuga.org/iuga/2014/39th/142301/ruchi.singh.perineal.z.plasty.for.introital.stenosis.a.video.presentation.html

## Figures and Tables

**Fig. 23:**
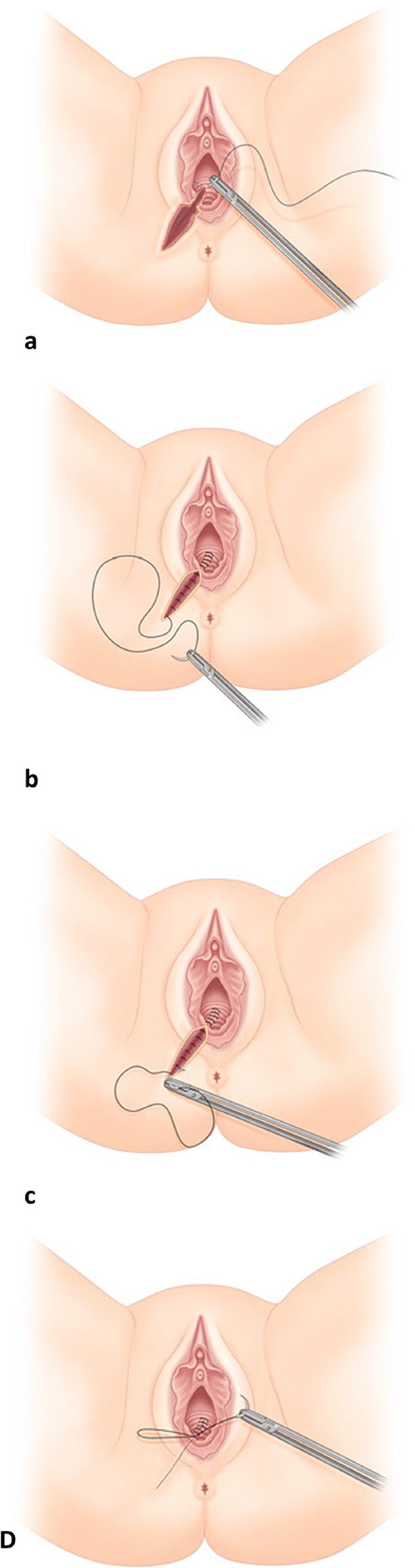
Episiotomy repair using continuous non-locking suture (all layers) [110]. (**a**) Episiotomy repair, suturing of vaginal wall. Insert first stitch to the vagina above the apex of episiotomy cut and tie a knot. (**b**) Episiotomy repair, suturing of perineal muscles. (**c**) Episiotomy repair, suturing of perineal skin. Starting from the bottom edge of the episiotomy cut, close perineal skin in the opposite direction towards the vaginal orifice using subcuticular continuous suture. (**d**) Episiotomy repair, knotting of the stitch. Place the subcuticular stitch in the vagina just above the level of the hymen and tie a knot. *Source:* Reprinted by permission from Springer Nature

## Appendix 1 - Methodological criteria for the selection of definitions

Potentially eligible terms for inclusion were identified and included into a term inventory. Subsequently each member of the WG completed a Delphi survey in order to prioritize the most relevant terms with a Likert type scale (1–7) from 1 (least relevant) to 7 (most relevant).

The terms with the highest scores were the subject of group discussion in order to decide a cut-off of relevance, with regards to pelvic floor anatomy and function changes during pregnancy, the puerperium and/or up to 12 months postpartum. Their definitions were reviewed and those already existing in other documents were considered for inclusion based on relevance to pregnancy, puerperium and postpartum period of 12 months. Some definitions were modified and new definitions were introduced where no previous definitions existed.

The following criteria were used to inform the inclusion and detail required to define each term:

1. Is this term childbirth trauma related?
2. Is this a separate entity to other PFD types (eg Postpartum stress urinary Incontinence vs generic SUI)?
3. Is this term covered by other Terminology documents?
4. Is this term covered or described in other literature?
5. Does this term need to be modified?
6. Is this term important or relevant enough to be repeated in this document if it features in other reports?
7. Is a footnote or appendix indicated/warranted for this term?
8. Does this term require an illustration?
9. Does this term require a video link?

## Appendix 2 - Repair of episiotomy

See Fig. 23.

**Surgical repair of vaginal and perineal trauma**

1. Repair of perineal and vaginal tears

- Following informed consent and adequate pain relief, the position of the woman should allow visualization of the vaginal and perineal tear. This usually involves lithotomy position.
- The first suture is applied above the apex of the vaginal component of the tear to ensure haemostasis.
- The vaginal part of the wound is sutured with a continuous, non-locking technique. This is associated with less pain and dyspareunia compared to interrupted sutures.
- The perineal muscles should be apposed and sutured with the same continuous suture, aiming to approximate the muscle such that the skin edges can be closed without tension. Two layers of continuous sutures may be required in deep tears.
- The perineal skin is usually closed with a continuous subcuticular suture.
- Following repair, a vaginal and rectal examination will ensure the repair is complete and there is no other trauma.

2 Repair of anal sphincter injuries [4]

- Repair of anal sphincter injuries should be undertaken by a clinician who is competent and ideally following formal training.
- Ideally these injuries should be repaired immediately after birth however there is no difference in functional outcome if the repair is delayed by a few hours e.g. because of lack of trained practitioner.
- The repair should take place in an operating theatre environment under regional or general anaesthesia for more complete examination and easier identification of the anatomy.
- A rectovaginal perforation (buttonhole tear) this should be repaired using two layers of interrupted polyglactin sutures.
- In the case of a fourth-degree tear, trauma to the anal epithelium should be repaired with interrupted 3/0 polyglactin sutures with the knots tied in the anal lumen.
- Any trauma to the internal anal sphincter should be repaired separately with interrupted horizontal mattress sutures using a fine suture such as 3/0 polydioxanone (PDS) or 2/0 polyglactin (Fig. 21). Separate identification and repair of the internal anal sphincter is associated with better continence outcomes.
- The torn ends of the external anal sphincter are held with Allis tissue forceps and sutured using either an overlap (if the muscle is completely torn, i.e. 3B/3C) or end-to end approximation, using 3/0 polydioxanone (PDS) or 2/0 polyglactin. When an overlapping technique is used, one or both sphincter ends may need to be dissected from surrounding tissue to provide sufficient length for the overlap. There is no difference in perineal pain, dyspareunia, fecal incontinence or flatal incontinence between the two techniques, although there is some evidence of a lower incidence of fecal urgency and lower anal incontinence symptom scores in the overlap group.
- Following repair of the sphincter, it is important to perform perineal reconstruction for support of the sphincter muscles.
- Repair of the vagina and perineum should proceed as for a second-degree tear.
- A rectal examination should be carried out to ensure that the repair is complete and that no sutures have been placed inadvertently through the rectal mucosa.
- An indwelling catheter should be left in the bladder for 12 to 24 h.
- Broad spectrum antibiotics at the time of the repair, plus oral antibiotics for 5 to 7 days after are recommended.
- Laxatives are recommended in the post-natal period to avoid constipation.
