# Correction to: An International Continence Society (ICS)/ International Urogynecological Association (IUGA) joint report on the terminology for the assessment and management of obstetric pelvic floor disorders

**DOI:** 10.1007/s00192-023-05507-3

**Published:** 2023-03-23

**Authors:** Stergios K. Doumouchtsis, Renaud de Tayrac, Joseph Lee, Oliver Daly, Joan Melendez-Munoz, Fiona M. Lindo, Angela Cross, Amanda White, Sara Cichowski, Gabriele Falconi, Bernard Haylen

**Affiliations:** 1 Department of Obstetrics and Gynaecology, Epsom and St. Helier University Hospitals NHS Trust, Epsom, UK; 2 St. George’s University of London, London, UK; 3 Laboratory of Experimental Surgery and Surgical Research “N.S. Christeas”, National and Kapodistrian University of Athens, Medical School, Athens, Greece; 4 School of Medicine, American University of the Caribbean, Cupecoy, Sint Maarten; 5 School of Medicine, Ross University, Miramar, FL USA; 6 Nimes University Hospital, Nimes, France; 7 University New South Wales, Sydney, Australia; 8 Western Health, Melbourne, Australia; 9 Hospital Universitari Dr. Josep Trueta, Girona, Spain; 10 Houston Methodist Hospital, Texas A&M University College of Medicine, Houston Methodist Hospital, Houston, TX USA; 11 Middlemore Hospital, Auckland, New Zealand; 12 University of Texas at Austin, Austin, TX USA; 13 Oregon Health & Sciences University, Portland, OR USA; 14 Complex Operative Unit of Gynecology, Fondazione Policlinico Tor Vergata University Hospital, Rome, Italy

**Correction to: International Urogynecology Journal (2022) 34:1-42**

10.1007/s00192-022-05397-x

The authors regret that one of the article references has been erroneously replaced by a non relevant one.

Reference 14 should be replaced by:

Haylen, B. T. et al. An International Urogynecological Association (IUGA) / International Continence Society (ICS) Joint Report on the Terminology for Female Pelvic Organ Prolapse (POP). Neurourol. Urodyn. (2016) 10.1002/nau.22922.

The authors would like to apologise for any inconvenience caused.

